# A synthetic lung model (ASYLUM) for validation of functional lung imaging methods shows significant differences between signal-based and deformation-field-based ventilation measurements

**DOI:** 10.3389/fmed.2024.1418052

**Published:** 2024-09-04

**Authors:** Andreas Voskrebenzev, Marcel Gutberlet, Filip Klimeš, Till F. Kaireit, Hoen-oh Shin, Hans-Ulrich Kauczor, Tobias Welte, Frank Wacker, Jens Vogel-Claussen

**Affiliations:** ^1^Institute for Diagnostic and Interventional Radiology, Hannover Medical School, Hannover, Germany; ^2^Biomedical Research in Endstage and Obstructive Lung Disease Hannover (BREATH), Member of the German Center for Lung Research, Hannover, Germany; ^3^Department of Diagnostic and Interventional Radiology, University Hospital of Heidelberg, Heidelberg, Germany; ^4^Translational Lung Research Center Heidelberg (TLRC), Member of the German Lung Research Center (DZL), Heidelberg, Germany; ^5^Clinic of Pneumology, Hannover Medical School, Hannover, Germany

**Keywords:** lung proton MRI, free-breathing, registration, phantom, Jacobian, Fourier decomposition, PREFUL

## Abstract

**Introduction:**

Validation of functional free-breathing MRI involves a comparison to more established or more direct measurements. This procedure is cost-intensive, as it requires access to patient cohorts, lengthy protocols, expenses for consumables, and binds working time. Therefore, the purpose of this study is to introduce a synthetic lung model (ASYLUM), which mimics dynamic MRI acquisition and includes predefined lung abnormalities for an alternative validation approach. The model is evaluated with different registration and quantification methods and compared with real data.

**Methods:**

A combination of trigonometric functions, deformation fields, and signal combinations were used to create 20 synthetic image time series. Lung voxels were assigned either to normal or one of six abnormality classes. The images were registered with three registration algorithms. The registered images were further analyzed with three quantification methods: deformation-based or signal-based regional ventilation (JVent/RVent) analysis and perfusion amplitude (QA). The registration results were compared with predefined deformations. Quantification methods were evaluated regarding predefined amplitudes and with respect to sensitivity, specificity, and spatial overlap of defects. In addition, 36 patients with chronic obstructive pulmonary disease were included for verification of model interpretations using CT as the gold standard.

**Results:**

One registration method showed considerably lower quality results (76% correlation vs. 92/97%, *p* ≤ 0.0001). Most ventilation defects were correctly detected with RVent and QA (e.g., one registration variant with sensitivity ≥78%, specificity ≥88). Contrary to this, JVent showed very low sensitivity for lower lung quadrants (0–16%) and also very low specificity (1–29%) for upper lung quadrants. Similar patterns of defect detection differences between RVent and JVent were also observable in patient data: Firstly, RVent was more aligned with CT than JVent for all quadrants (*p* ≤ 0.01) except for one registration variant in the lower left region. Secondly, stronger differences in overlap were observed for the upper quadrants, suggesting a defect bias in the JVent measurements in the upper lung regions.

**Conclusion:**

The feasibility of a validation framework for free-breathing functional lung imaging using synthetic time series was demonstrated. Evaluating different ventilation measurements, important differences were detected in synthetic and real data, with signal-based regional ventilation assessment being a more reliable method in the investigated setting.

## 1 Introduction

Functional proton lung MRI gained interest during the last few years, as it allows to assess lung function in free-breathing without ionizing radiation, inhalation of gases, or contrast agent application (1). These methods can be divided into two basic groups: signal-based and deformation-based. While both approaches require image registration to account for diaphragmatic and cardiac motion, the first derives ventilation and perfusion parameters from the signal values, and the latter uses the geometric information to derive ventilation based on the calculated expansion/shrinkage of voxels during registration.

Currently, the signal-based approach is more widespread, including Fourier decomposition (2), matrix pencil decomposition (3), self-gated non-contrast-enhanced functional lung (SENCEFUL) (4, 5), and phase-resolved functional lung (PREFUL) MRI (6, 7). Nevertheless, some studies showed promising results in 2D and 3D with the deformation-field-based approach (8–11). Since both approaches are surrogates, in addition to mandatory reproducibility measurements (12–15), it is also necessary to perform extensive validation with well-established reference methods such as inhaled gas MRI for ventilation or dynamic contrast-enhanced MRI for perfusion (16–20). This process requires access to patient cohorts, additional hardware (hyperpolarization, multinuclear RF coils), consumables, extended MRI protocols, manpower, and costly scanner time. Furthermore, it is not expected that inhaled gas MRI ventilation measurement performed in breath-hold will fully coincide with a free-breathing method purely due to physiologic reasons (21). Similarly, while correlations were observed in DCE and perfusion-weighted signal-based measurements, it is important to note that these methods rely on fundamentally different mechanisms. DCE measures signal changes due to T1 shortening, which is induced by the transport pathway of contrast media concentration. In contrast, signal-based methods measure the time-of-flight effect of unsaturated blood spins entering the slice. Additionally, patient compliance might limit the practicability in certain cases and might lead to increased variability (22).

This motivates us to approach the problem from a different perspective. Similar to conducting phantom measurements during sequence design, a synthetic lung model (ASYLUM) may be used as a complementary validation tool for post-processing algorithms. By applying known regional deformation and using model functions for signal time-series generation, different lung function states can be simulated and made directly available as a gold standard.

Therefore, the aim of this study is to describe a method to create ASYLUM and utilize this model by evaluating different registration and lung function quantification methods. Although it is possible to evaluate registrations by comparing structure alignment and other image similarity metrics (23), ASYLUM includes a known deformation for direct registration evaluation. The signal-based regional ventilation (RVent) (24) and deformation-field-based Jacobian determinant (JVent) (8) were selected to test whether the theoretical equivalence of both methods can be verified. Additionally, a simple implementation of a signal-based perfusion-weighted amplitude (QA) measurement was included to account for the perfusion abnormalities, which are also modeled in ASYLUM. Finally, all the described ventilation methods were evaluated in a patient cohort to see if the findings were also confirmed by real data using CT as a gold standard.

## 2 Theoretical considerations

### 2.1 Basic theory of signal-based pulmonary functional MRI

As described previously (25), the pulmonary time series of MR signal acquired with a fast spoiled gradient echo sequence during free-breathing can be described by four main components: proton density, time-of-flight (TOF) effect, movement, and noise. This is an incomplete model (e.g., T1, T2/T2* decay, diffusion, field inhomogeneities, direction of flow, artifacts), but previous validation studies (16–19, 26) suggest that these factors probably only play a minor role with proper imaging protocols and reconstructions (e.g., minimal TR in combination with low flip angles to achieve proton density (PD) weighting, asymmetric echo to reduce TE and hence T2* decay and further propel PD weighting, T-GRAPPA to exploit the dynamic nature of the acquisition, and apply surface coil intensity correction to avoid artificial regional signal variations). Therefore, for simplicity, these influences are omitted from the following description.

The variability due to movement is compensated by image registration, which will be discussed in the next section. During expiration, the decreasing lung volume leads to an increased proton density and therefore increased MR signal, and vice versa during inspiration. Therefore, proton density is a function of respiration, and the inverse relationship of signal and volume is the foundation for ventilation measurements (27).

The continuous application of excitation pulses over a short period of time leads to incomplete relaxation, which finally converges to a steady state. Inflowing spins have not reached this state and have a higher initial signal in comparison with stationary spins. Thus, the pulsatile inflow of blood leads to further signal variation, also known as TOF, and is the foundation for perfusion measurement.

Due to the inherent low signal of the lung (28), averaging and filtering are required to achieve a sufficient signal-to-noise ratio (SNR). Ideally, after registration, averaging, and filtering, the variation of MR signal contains only information about ventilation and perfusion. Since these variations occur at different frequencies, they can be individually evaluated by Fourier analysis (29).

Using the inverse relationship, a relative volume change can be quantified as RVent (24), which is a generalized version of fractional and specific ventilation accounting for the registered volume:


(1) $$RVent=\frac{v_{Insp}-v_{\exp}}{v_{Reg}}=\frac{1/s_{Insp}-1/s_{\exp}}{1/s_{Reg}}=\frac{s_{Reg}\left(s_{\exp}-s_{Insp}\right)}{s_{Insp}s_{\exp}}$$

with inspiration (Insp), expiration (Exp), and registration (Reg) volumes (v) and signals (s). The registration volume is the final volume after registration, also denoted as a fixed image.

Quantification of perfusion is more complex as it involves the estimation of blood and exchange fractions (30, 31). Since the main concern of this study is the accurate detection of ventilation and perfusion defects, only a perfusion-weighted measurement was implemented in this study by estimating the amplitude of the TOF signal variation component.

The practical implementation of the described methods is described in a later section.

### 2.2 Basic theory of pulmonary deformation-based ventilation measurement

Although the registration process cannot be completely independent of signal values, which constitute the respective image, the ventilation result of deformation-based ventilation measurements is ultimately derived from geometric information. The deformation field includes the displacement vectors to map the voxels from the moving volume to the fixed volume.

The partial derivatives of this deformation field will reveal local expansion and deflation. For intuitive comprehension, the following extreme cases can be considered: If all voxels can be described by an arbitrary but constant displacement, no local expansion or inflation is present, and the derivatives will be zero. If instead only one voxel is displaced (or “copied”) by one voxel, the partial derivative in this direction at this location will be 100% and corresponds rightly to an expansion factor of 2, since the voxel expanded its size from one voxel to two voxels.

Typically, the total displacement *f* is described as the sum of the identity and registration displacement operation. Then, the total expansion/deflation calculation is mathematically described as the determinant of the Jacobi matrix. The Jacobi matrix includes all partial derivatives of the total displacement *f*, and the determinant can be interpreted as the area/volume parallelogram. Thus, the ventilation with the Jacobi method (JVent) can be calculated as follows:


(2) $$JVent=\frac{v_{Insp}-v_{\exp}}{v_{Reg}}=\det\left(J_{f}\right)-1$$

## 3 Materials and methods

### 3.1 Subjects

To validate the derived results from the model data, a subset of data from the COSYCONET study (32), assessing patients with chronic obstructive pulmonary disease (COPD), was carried out retrospectively [*n* total = 36, *n* female = 21, median age 63.5, age range 42–77, GOLD I (*n* = 10), II (*n* = 9), III (*n* = 12), IV (*n* = 5)].

The study inclusion criteria were as follows: COPD- or chronic bronchitis-diagnosed patients aged 40 or older years with availability for repeated study visits over at least 18 months.

The study exclusion criteria were as follows: major lung surgery, moderate or severe exacerbation within the last 4 weeks, presence of lung tumor, inability to walk or understand the intention of the project.

Baseline scans conducted between 16 February 2011 and 4 December 2013 at the Hannover site were included in this subset, comprising completed CT and MRI scans. COSYCONET was conducted in accordance with the Declaration of Helsinki and Good Clinical Practice Guidelines. Approval was obtained from the ethics committees of the participating centers and from the concerned data security authority. All participants provided written informed consent.

### 3.2 Imaging procedure

#### 3.2.1 MRI

Acquisition was performed on a 1.5 T system (Avanto, Siemens Healthineers, Erlangen, Germany) using a spoiled gradient echo sequence with the following settings provided as median with range (in cases of variability) in parenthesis: field-of-view (FOV) 500 × 500 mm^2^, echo time (TE) 0.82 (0.65–0.82) ms, repetition time (TR) 3 ms, temporal resolution 288 (192–288) ms, slice thickness 15 mm, matrix 128 × 96 (128–64 − 128-128), parallel imaging acceleration factor with T-GRAPPA (33) 1 (1–2), receiver bandwidth (BW) 1,500 Hz/Pixel for a total of 200 (200–250) image frames for each slice. A total of four coronal slices were acquired in free-breathing for each patient, with the reference slice positioned at the tracheal bifurcation and two slices in the posterior and one in the anterior direction. The inter-slice distance was set to 3 mm (20%).

#### 3.2.2 CT

Patients were scanned in supine position with a 64-slice scanner without intravenous contrast media with the following settings: tube current 120 kV, automatic tube modulation, table feed 39.375 mm/gantry rotation, 0.625 mm slice thickness, and 0.7 mm reconstruction interval using a “standard” reconstruction kernel.

Scans were performed in full in- and expiration to capture the lung in total lung capacity and residual volume state.

### 3.3 ASYLUM—procedure outline

The creation of ASYLUM is a 3-fold procedure:

1. Create a base geometry, which is used as a starting point, and assign voxel classes to reflect different tissues present in the image (e.g., normal and abnormal lung voxels).
2. Change the geometry according to local expansion factors to reflect local expansion and deflation.
3. Fill the geometry with appropriate signals and noise, which reflect MR physics and are in concordance with the geometry change and voxel class defined in the first and second steps.

**Figure 1:**
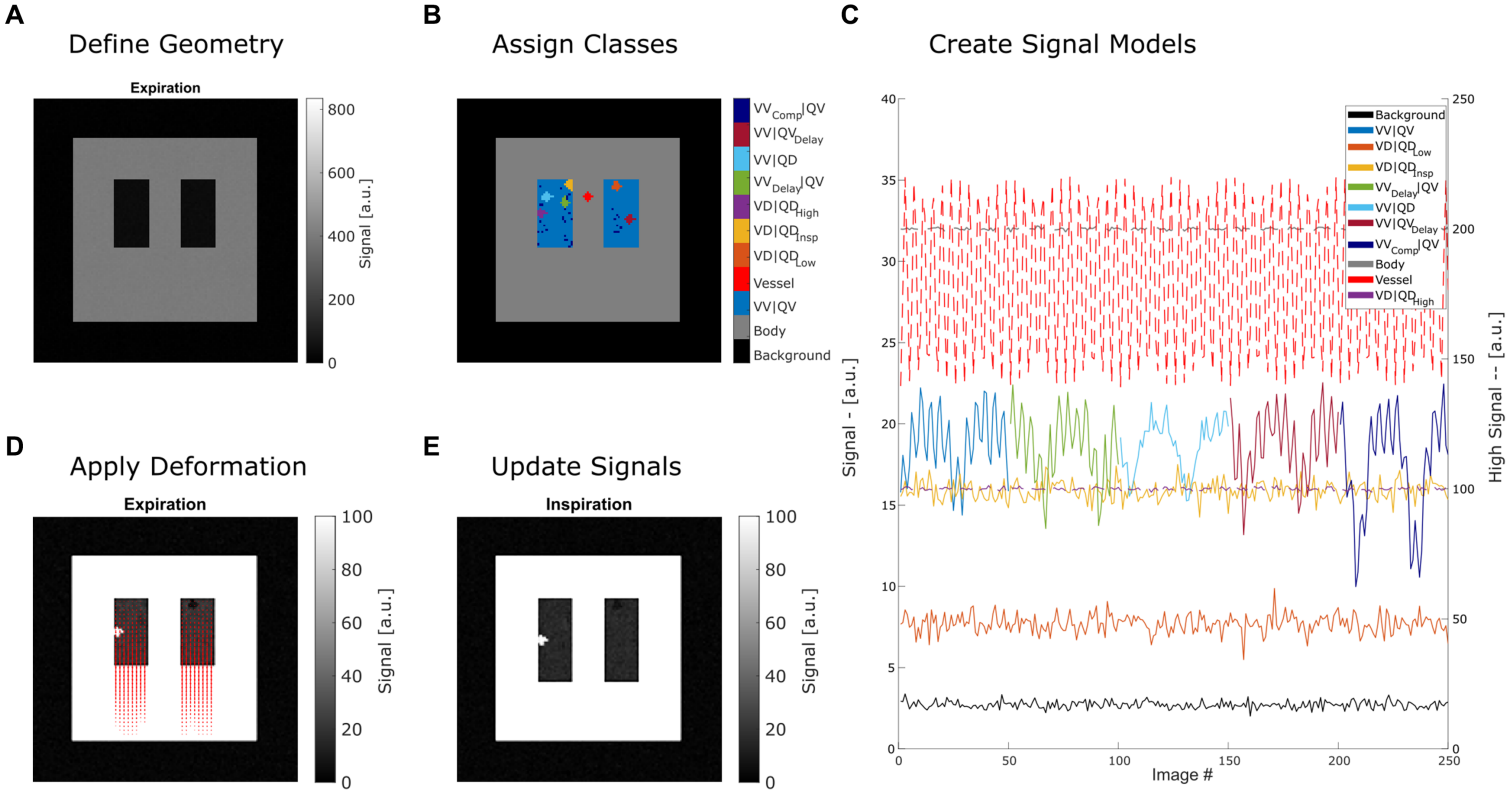
Summary of creating a synthetic lung model (ASYLUM) to mimic free-breathing MRI data. The steps consist of defining a simplified coronal lung anatomy **(A)**, random assigning of classes **(B)** for each lung voxel, application of deformation according to respiratory state and local expansion **(D)**, and thus creating an image time series **(E)** with respective time series **(C)**. Please note that for display purposes, some classes were displayed with truncated time series in **(C)**, and two axes were used (dashed line indicates that the high signal axis on the right was used). VV|QV: ventilated and perfused volume, VD|QD_Low_: ventilation and perfusion defect below inspiration signal level, VD|QD_Insp_: ventilation and perfusion defect at inspiration level, VD|QD_High_: ventilation and perfusion defect at high signal level, VV_Delay_|QV: ventilated and perfused volume with delayed ventilation, VV|QD: ventilated volume with perfusion defect, VV|QV_Delay_: ventilated and perfused volume with delayed perfusion.

Steps 2 and 3 can be repeated with different expansion factors to create a time series of images. By changing the classes in step 1, variations of the model can be created. These three steps are described in detail in the following sections and are illustrated in Figure 1.

To reduce inaccuracies, especially during step 2, the calculations were performed on 4-fold upscaled data. Afterwards, the data were scaled back to original size. Before further processing, to mimic a typical PREFUL or Fourier decomposition acquisition, the data were once again interpolated to 256 × 256 matrix (factor 2).

### 3.4 ASYLUM—basic model and voxel classes

The initial 2D coronal lung morphology in expiration was constructed by three rectangles in a 128 × 128 matrix. This defines the three basic classes (1): the body (outer border 90 × 90) (2), the right and left lung (16 × 32 each), and (3) the remaining voxels as background (pure noise). Additionally, between both lungs, a vessel was defined as fourth class using a disk geometry with a radius of two voxels.

The lung class is further classified into eight subclasses:

1. Normally ventilated and perfused voxels (VV|QV) with expansion factor e
2. Ventilation and perfusion defect at constant (independent of respiration phase) signal corresponding to the halved value of normal lung parenchyma during inspiration phase (VD|QD_Low_) with e = 0 (e.g., emphysema)
3. Ventilation and perfusion defect with constant (independent of respiration phase) signal with a value corresponding to the value of normal lung parenchyma during inspiration phase (VD|QD_Insp_) with e = 0 (e.g., air trapping)
4. Ventilation and perfusion defect with constant (independent of respiration phase) signal with a value corresponding to the 5-fold value of normal lung parenchyma during expiration level (VD|QD_High_) with e = 0 (e.g., infiltrate)
5. Delayed ventilation and normally perfused voxels (VV_Delay_|QV) (e.g., early disease manifestation) with expansion factor *e*
6. Ventilated perfusion defect (VV|QD) (e.g., V/Q mismatch) with expansion factor e
7. Voxel with normal ventilation but delayed perfusion (VV|QV_Delay_) (e.g., early disease manifestation) with expansion factor *e*
8. Higher ventilated (compensatory) and normally perfused voxels (VV_Comp_|QV) with expansion factor *e_compensate_.*

The subclassification is performed using a randomized seed placement, which will result in different cluster locations for each class for repeated model generation. The size of each defect class was set to 60 voxels, equivalent to 6% of the expiratory lung volume (1024).

### 3.5 ASYLUM—warping

Since ASYLUM must reflect an acquisition in free-breathing, one main challenge is the modeling of respiratory movement. This can be achieved by inverting the registration problem, which tries to compensate movement. Therefore, by definition of adequate deformation, movement can be simulated. For simplicity, the respiratory movement was assumed to be one-dimensional (*y*-direction). This movement can be mathematically described by a deformation field *F*_*x,y*_, which includes the amount of *y*-displacement for each voxel. To find this deformation field, an auxiliary matrix *E* containing all local expansion factors *e* was created according to the assigned lung classes (see previous section). This will initially result in unbalanced expansions, since some columns (*y*-direction) will include ventilation defects with no expansion. In addition, lung expansion must be compensated by the shrinking of the body class. Therefore, lung compensation voxels and body voxels were assigned with expansion factors to meet the following conditions:


(3) $$C_{1}=\frac{1}{32}\overset{\mathrm{Lung Start}+31}{\underset{y=\mathrm{Lung Start}}{\sum }}E_{x,y}=e\forall x$$

$$C_{2}=\overset{127}{\underset{y=0}{\sum }}E_{x,y}=0\forall x$$

The first condition, *C*_1_, will ensure that the average lung expansion factor e is uniform (1.25) along all columns of the lung. The second condition, *C*_2_, will establish adequate deflation of the body so that the overall expansion factor is one. Please note that 32 is the lung size in *y*-direction defined previously.

The deformation field in expiration space (forward deformation *F_x,y_*) is obtained by applying the cumulative sum operation on the *E_x,y_* matrix in *y*-direction. Since warping is typically performed with inverse deformation fields (deformed or fixed space) to avoid problems of “holes” and both fields will be required anyway to simulate a “perfect registration,” the inverse deformation field was calculated by performing a linear scattered interpolation on the irregular transformed grid (*i,j*) defined by the forward deformation:


i=x+0=x

$$j=y+E_{x,y}$$

Please note that there is no deformation in *x*-direction. Finally, the scattered interpolant S was evaluated at a regular grid in deformed space and reversed in direction to obtain the inverse deformation field *I_x,y_*:


$$I_{x,y}=-S\left(x,y\right)$$

Thus, to obtain an image geometry *G*(*e*) according to an expansion factor *e*, the expiration image is warped with *I_x,y_*. A pseudo registration of this image would be performed by a consecutive image warping ( • ) with the forward deformation *F_x,y_*, which will in theory give the initial geometry of the expiration image. Therefore, all required deformation operations can be summarized as follows:


$$G\left(e\right)=G\left(1.0\right)•I_{x,y}$$

$$G\left(1.0\right)=G\left(1.0\right)•I_{x,y}•F_{x,y}=G\left(e\right)•F_{x,y}$$

### 3.6 ASYLUM—signal time-series generation

To fill the geometry with different signal time series *s*(*t*), the Lujan et al. (34) formula, as modified by Bauman et al. (2) was used:


$$s\left(t\right)=s_{0}-A_{V}\cos^{2n}\left(\frac{\pi t}{\tau_{V}}-\varphi_{V}\right)+A_{Q}\sin^{2m}\left(\frac{\pi t}{\tau_{Q}}-\varphi_{Q}\right),$$

with the ventilation (*V*) and perfusion (*Q*) signal amplitudes *A*, periods τ and phases φ and signal shape parameters *n* and *m*. The following parameters were set according to the provided example of Bauman et al.: $n=3,m=2,\tau_{V}=5\;s,\tau_{Q}=0.8.$ The remaining parameters were adapted to fit the respective voxel class with arbitrarily set base signal levels $s_{\exp}=20\;AU,s_{Body}=200\;AU$ :

1. VV|QV: $\begin{array}{l} e=0.25;s_{0}=s_{\exp};A_{Q}=6;A_{V}=s_{\exp}•\left(1-1/\left(1+e\right)\right);\\ \varphi_{V}=0;\varphi_{Q}=0 \end{array}$ ; the last two settings indicating no delay in ventilation and perfusion
2. VD|QD_Low_: The same as first class, but with perfusion amplitude $A_{Q}=0$ , expansion factor e=0 and $s_{0}=s_{Insp}/2$
3. VD|QD_Insp_: The same as second class, but with $s_{0}=s_{\exp}/\left(1+e_{Ventilated}\right)=s_{\exp}/1.25=16\;AU$
4. VD|QD_High_: The same as second class, but with $s_{0}=s_{\exp}•5=100\;AU$
5. VV_Delay_|QV: The same as first class, but with $\varphi_{V}=\pi /2$
6. VV|QD: The same as first class, but with $A_{Q}=0$
7. VV|QV_Delay_: The same as first class, but with $\varphi_{Q}=\pi /2$
8. VV_Comp_|QV: The same as first class, but with increased expansion factor e_Compensate_ as defined in Equation 3.

The body and background class were assigned with a fixed signal as they contained no dynamic component. The vessel class was assigned with the following parameters: $s_{0}=s_{Body}\cdot 0.9;A_{Q}=120;A_{V}=0;\varphi_{V}=0;\varphi_{Q}=\pi /2.$

Finally, an artificial sum-of-squares coil combination was performed to simulate MR signals with adequate noise distributions (35). For this purpose, the normally distributed noise of a pseudo-four-channel coil was added to the signal *s*(*t*) with a standard deviation set in such a way, that VV voxels ended up with a SNR of 5.

To produce a time series of images, the expansion factor was modulated according to the ventilation phase during the specific time point. For this purpose, the inverse of the signal time series was used as a surrogate for volume *v*(*t*,*x*) (27), from which the respiration factor *r*(*t*,*x*) was derived to scale the expansion factor according to volume:


$$v\left(t,x\right)=1/\left(s_{0}-A_{V}\cos^{2n}\left(\frac{\pi t}{\tau_{V}\left(x\right)}-\varphi_{V}\left(x\right)\right)\right)$$

$$e\left(t,x\right)=e\left(x\right)\cdot \;r\left(\mathrm{t,x}\right)=e\left(x\right)\cdot \;\frac{v\left(\mathrm{t,x}\right)-\min\left(v\left(\mathrm{t,x}\right)\right)}{\max\left(v\left(t,x\right)\right)-\min\left(v\left(t,x\right)\right)}$$

Please note that the respiration factor r(t,x) is used to scale *e*(*t*,*x*) between 0 and *e*(*x*) according to the respective volume during the time point t.

For the final creation of an image time series, deformation was performed according to *e*(*t*,*x*), and the geometry was filled as described in this section.

The time was parametrized with 250 entries using an increment of 0.192 s (temporal resolution).

### 3.7 Registration and ventilation imaging

Non-rigid motion correction was performed with four methods with a one-step registration using the expiration state as a fixed image:

1. Reference registration (REF) by using the forward deformation field as described in the ASYLUM-warping section. The only error source in this case is the transformation between forward and inverse deformation fields.
2. Advanced normalization tools (ANTs) (36), which were successfully used in multiple previous studies (14, 37, 38). The b-spline model with cross-correlation as a similarity metric was used. Denoted as ANTs in the following.
3. The Forsberg registration package (39, 40) with polynomial expansion method and normalized cross-correlation and mean square error as metric, which was recently shown to deliver similar quality to ANTs in the context of 3D PREFUL paired with a potential speed advantage (41). Denoted as F-REG in the following.
4. A diffeomorphic demons algorithm (42) with accumulated field smoothing is implemented as an official function “imregdemons” in MATLAB (R2020b). Denoted as M-REG in the following.

For the patient data, only algorithms 2–4 were feasible.

Prior to registration, the respiration factor *r*(*t*) of the normal voxels was binned into 10 groups, each containing 10% of the data (25 images). Registration was performed toward the average expiration image.

The registered expiration and inspiration images (expiration and inspiration states according to VV|QV class) were averaged, and RVent was calculated according to Equation 1.

The deformation fields of the inspiration were averaged and the JVent was calculated according to Equation 2.

Due to low SNR or reduced functional signal amplitude, for the patient data, an image-guided edge-preserving filter was applied prior to RVent calculation (43) (the filtered result is denoted as RVent*). Otherwise, the processing was the same as for the ASYLUM data.

### 3.8 ASYLUM perfusion imaging

To assess the perfusion-related aspects of ASYLUM, perfusion-weighted analysis was performed as follows:

1. The registered image time series was high-pass filtered with a cutoff frequency of 0.9 Hz.
2. Phases with maximal and minimal signals were determined from the averaged signal in the lung ROI.
3. Perfusion-related amplitude (QA) was calculated as the difference between the maximal and minimal signal in lung parenchyma: $QA=s_{\max}-s_{\min}$ .

### 3.9 Ventilation and perfusion defect (VD/QD) analysis

As published in recent studies (14, 44), the VD and QD were defined as regions with values below a threshold determined from the respective 90th-percentile value multiplied by 0.4. VD/QD percentage (VDP/QDP) was defined as the number of voxels below the threshold in relation to the total number of voxels in the lung ROI.

### 3.10 Parametric response mapping

CT data were analyzed with parametric response mapping (PRM) (45). For this end, registration of inspiration toward expiration was performed as follows:

1. Semiautomatic segmentation of lung lobes by applying a local, adaptive region growing algorithm in inspiration and expiration with a dedicated software (MeVisPULMO 3D, Fraunhofer MEVIS Bremen, Germany).
2. Downsampling the image resolution in *x*- and *y*-direction by a factor of 2.
3. Non-rigid registration with ANTs.
4. Labeling of lung voxels as normal, voxels with functional small airways disease (fSAD) or emphysema according to Galban et al. (45).

To allow a regional comparison with MRI, appropriate slice positions were identified. To find an adequate transformation between the coordinates of MR and CT measurements, a landmark (tracheal bifurcation) was manually identified on the anatomical 3D MRI scan (used as a localizer) and CT. Using transformed coordinates, the coronal slice locations were identified in the CT data and averaged to produce corresponding 15-mm slices, as in MR. Then CT was registered toward MRI expiration state using only mask information of CT and MRI with non-rigid registration by ANTs. The obtained transformation was subsequently applied to PRM map. The three-class PRM map was simplified to a binary VD map by combining fSAD and emphysema class into one VD class.

### 3.11 Image and statistical analysis

Raw registration performance of ANTs, F-REG, and M-REG on ASYLUM data was assessed with three quantitative metrics using REF as a reference:

1. Root mean squared relative error (RMSRE) of the inspiration state registered to the expiration state calculated inside the lung ROI of the expiration state:

$$RMSRE=\sqrt{\frac{1}{n}\overset{n}{\underset{i=1}{\sum }}{\left|\frac{REG-REF}{REF}\right|}^{2}},$$

With *n* being the number of samples in the lung ROI, REG the registered inspiration image with the respective registration method (46).

2. Pearson correlation of the *y*-profile of the respective registration to the *y*-profile of the reference
3. Registration time.

To obtain regional information for certain statistics, the whole lung ROI was divided into the following equally sized quadrants: upper right (UR), upper left (UL), lower right (LR), and lower left (LL).

Averaged whole lung ROI values of RVent, JVent, and QA were used to calculate median and interquartile range values for all registration methods of the repeated experiment and visualized as boxplots. For regional comparison, the mean and median of the relative difference to the defined functional value were determined quadrant-wise.

Quadrant-wise true positive (sensitivity) and true negative rate (specificity) were determined for the binary measurements VD and QD based on RVent, JVent, and QA. Sensitivity was also evaluated for the individual defect classes. For this purpose, the quadrant ROIs were modified by additional masking of the respective non-involved defect classes. This was necessary, as the VD and QD did not further differentiate between different kinds of defects, and therefore an evaluation of the whole ROI would lead to a bias in the measurement.

For the comparison of patient data, median and interquartile range values were determined for averaged lung ROI values. Additionally, VD and total overlap derived from RVent and JVent in relation to CT VD were compared quadrant-wise.

Statistical differences between registration and ventilation measurement methods were tested with Friedman’s test as omnibus test. For *post-hoc* Wilcoxon signed rank test was performed at the 5% significance level.

## 4 Results

### 4.1 ASYLUM data

**Figure 2:**
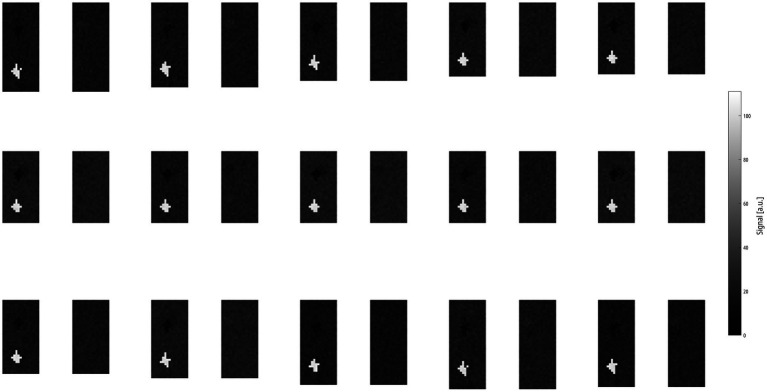
Montage of 15 ASYLUM frames sorted in left-to-right/top-to-bottom direction, skipping every second frame to better illustrate the respiration. Please also note the defect classes VD|QD_Low_ and VD|QD_High_, both located in the right lung, which are discernable due to signal contrast and exhibit no change in size. Please note that this is a zoomed-in view, not showing the background or parts of the body to better display the lung parenchyma. ASYLUM: a synthetic lung model, VD|QD_Low_: ventilation defect, perfusion defect below inspiratory signal level, VD|QD_High_: ventilation defect, perfusion defect with high signal.

Dynamic data were successfully created as described in the Methods section (see Figure 2 for examples of time frames obtained with ASYLUM).

#### 4.1.1 Registration performance

Significant but minor differences were found for the RMSRE metric for ANTs and the other registration algorithms (ANTs: 0.30, F-REG 0.27, and M-REG 0.27, *p* ≤ 0.05). Correlation differences of the *y*-displacement profile were more pronounced, with F-REG showing the best result at 97% vs. 92% (ANTs) and 77% (M-REG), *p* ≤ 0.0001. Similarly, the required registration time was significantly different between the algorithms: ranging from 150 min (ANTs) to 20 min (M-REG) and 7 min (F-REG). See Table 1, Part A, for a summary of all results.

**Table 1:** (A) Median and interquartile range values of registration performance metrics: root mean squared relative error (RMSRE) using the reference registration, Pearson correlation of the *y*-displacement profile, and registration time. (B) Median and interquartile range values of RVent, JVent, and QA parameters for all registrations and respective omnibus and *post-hoc* test results for difference.

**(A)** Registration	RMSRE	Correlation (%)	Registration time (min)
ANTs	0.30 (0.27–0.35)	91.91 (91.56–93.07)	149.84 (110.28–151.29)
F-REG	0.27 (0.26–0.28)	97.18 (96.84–97.37)	7.19 (5.71–9.48)
M-REG	0.27 (0.26–0.29)	76.64 (75.12–78.04)	19.64 (19.25–20.82)
Omnibus	1.06e−02 (*)	2.06e−09 (****)	5.33e−09 (****)
ANTs vs. F-REG	3.59e−03 (**)	8.86e−05 (****)	8.86e−05 (****)
ANTs vs. M-REG	1.11e−02 (*)	8.86e−05 (****)	1.03e−04 (***)
F-REG vs. M-REG	7.65e−01 (n.s.)	8.86e−05 (****)	8.86e−05 (****)

**(B)** Functional values	RVent	JVent	QA	RVent vs. JVent
REF	0.24 (0.24–0.24)	0.24 (0.24–0.24)	3.99 (3.99–4.01)	2.82e−03 (**)
ANTs	0.39 (0.35–0.55)	0.24 (0.23–0.24)	3.94 (3.92–3.96)	8.86e−05 (****)
F-REG	0.23 (0.23–0.23)	0.24 (0.24–0.24)	3.26 (3.24–3.30)	8.86e−05 (****)
M-REG	0.23 (0.23–0.23)	0.27 (0.26–0.27)	3.60 (3.54–3.64)	1.20e−04 (***)
Omnibus	2.21e−11 (****)	3.88e−12 (****)	5.88e−13 (****)	
REF vs. ANTs	8.86e−05 (****)	8.86e−05 (****)	8.86e−05 (****)	
REF vs. F-REG	8.86e−05 (****)	4.05e−03 (**)	8.86e−05 (****)	
REF vs. M-REG	1.51e−03 (**)	8.86e−05 (****)	8.86e−05 (****)	
ANTs vs. F-REG	8.86e−05 (****)	8.86e−05 (****)	8.86e−05 (****)	
ANTs vs. M-REG	8.86e−05 (****)	8.86e−05 (****)	8.86e−05 (****)	
F-REG vs. M-REG	5.50e−01 (n.s.)	8.86e−05 (****)	8.86e−05 (****)	

*Post-hoc* tests are denoted with an asterisk indicating significance level: *p* ≤ 0.05 (*), *p* ≤ 0.01 (**), *p* ≤ 0.001 (***), *p* ≤ 0.0001 (****). Please note that the numbers displayed in this table are rounded to two decimal places. As a result, different values may appear identical despite minor differences in their actual values. RMSRE: root mean squared relative error, REF: reference (known) registration, ANTs: advanced normalization tools, F-Reg: Forsberg registration, M-Reg: Matlab registration, RVent: regional ventilation, JVent: Jacobian determinant ventilation, QA: perfusion amplitude.

**Figure 3:**
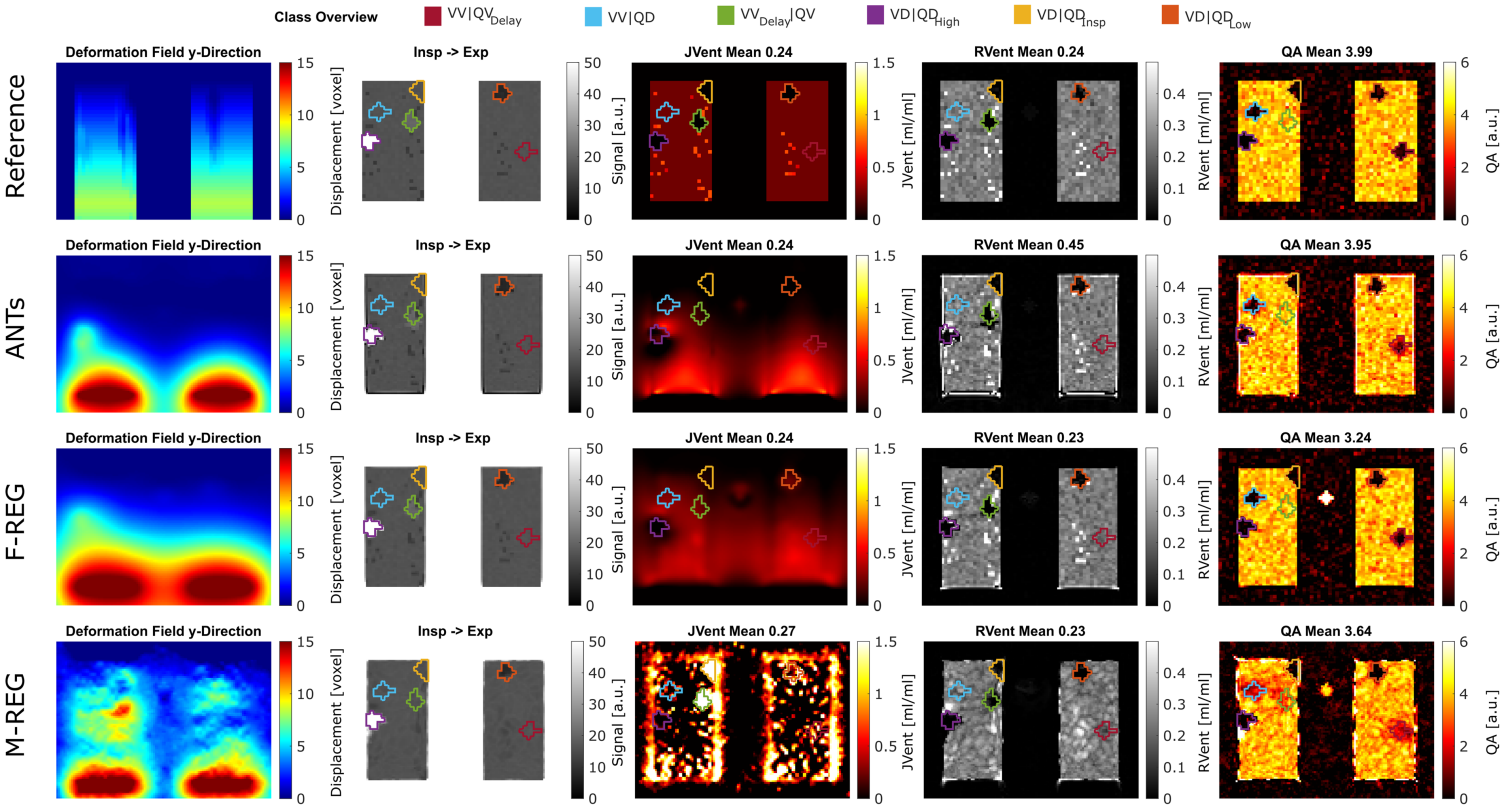
Exemplary performance of different registration and quantification methods for ASYLUM. For registration, reference (known deformation), advanced normalization tools (ANTs), Forsberg (F-REG), and Matlab (M-REG) algorithms were used (deformation fields in the *y*-direction are displayed in the first column). The registered image is displayed together with the color-coded class definitions in the second column. For ventilation, the Jacobian determinant (JVent, third column) and signal-based method (RVent, fourth column) were used. Additionally, a signal-based perfusion amplitude quantification (QA) is displayed in the last column. RMSRE: root mean squared relative error, ANTs: advanced normalization tools, F-Reg: Forsberg registration, M-Reg: Matlab registration, RVent: regional ventilation, JVent: Jacobian determinant ventilation, QA: perfusion amplitude, VV|QV: ventilated and perfused volume, VD|QD_Low_: ventilation and perfusion defect below inspiration signal level, VD|QD_Insp_: ventilation and perfusion defect at inspiration level, VD|QD_High_: ventilation and perfusion defect at high signal level, VV_Delay_|QV: ventilated and perfused volume with delayed ventilation, VV|QD: ventilated volume with perfusion defect, VV|QV_Delay_: ventilated and perfused volume with delayed perfusion.

Visual analysis (see example in Figure 3) confirmed the correlation results: M-REG showed clearly wrong displacement patterns and JVent maps, whereas ANTs and F-REG resulted in what seemed to be blurred versions of the REF displacement with an artificial increase of deformation from cranial to caudal as evident from the JVent maps.

#### 4.1.2 Functional parameters

Although significantly different, REF and F-REG resulted in whole lung mean RVent and JVent values approximating the expected value of 0.25 within a margin of 0.02 or less for the values within IQR. Both REF and ANTs were nearly at the expected four arbitrary units (a.u.) mark for QA: 3.99 (REF) and 3.94 (ANTs). Contrary to this, F-REG and M-REG were off by a significantly higher margin: 3.26 (F-REG) and 3.60 (M-REG). See Table 1, Part B, for a summary of all results.

**Figure 4:**
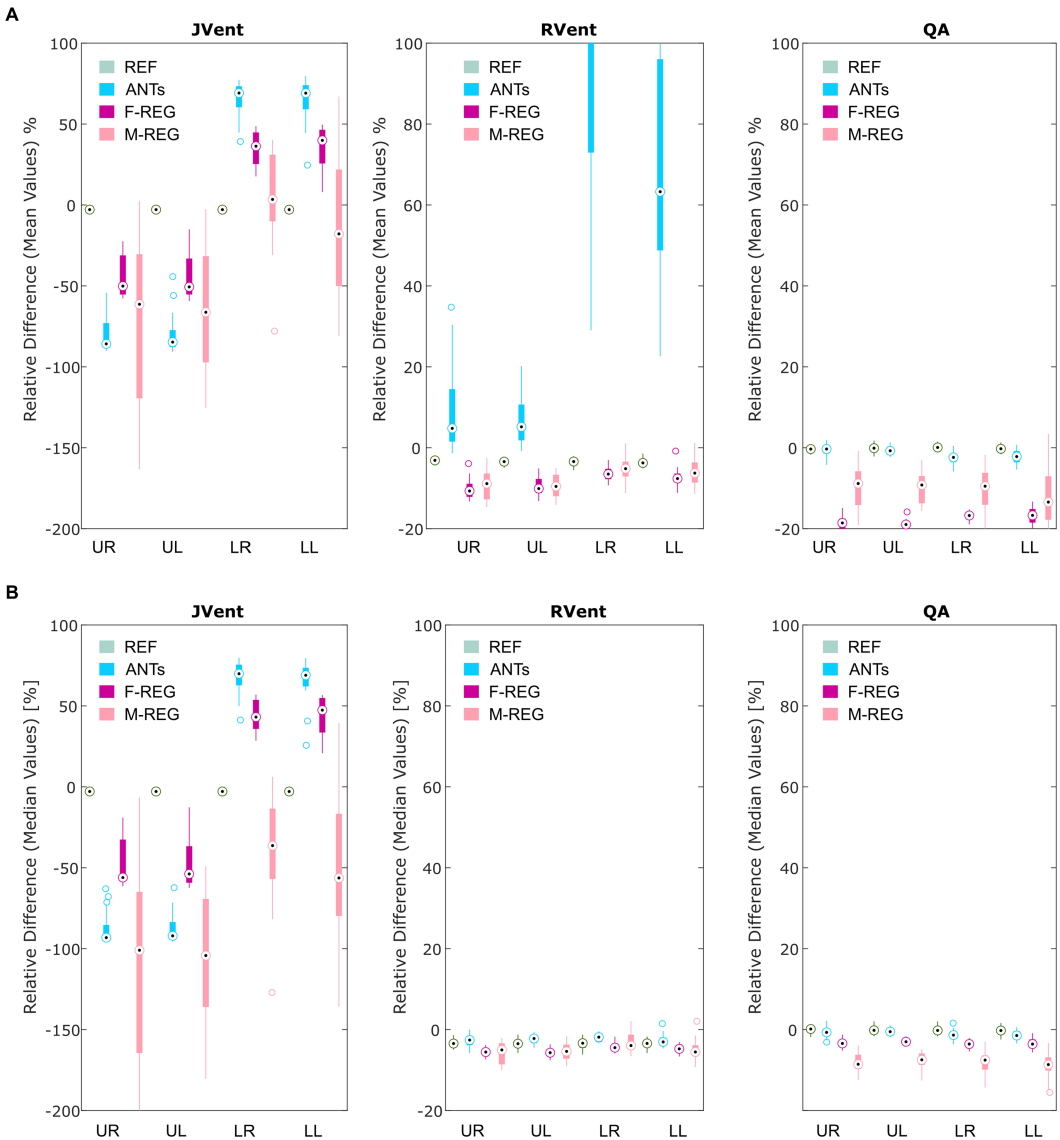
Boxplots of the mean **(A)** and median **(B)** relative differences in relation to the predefined functional values for all registration variants depending on quadrant. REF: reference (known) registration, ANTs: advanced normalization tools, F-Reg: Forsberg registration, M-Reg: Matlab registration, RVent: regional ventilation, JVent: Jacobian determinant ventilation, QA: perfusion amplitude, UR: upper right, UL: upper left, LR: lower right, LL: lower left.

Visual inspection (see example in Figure 3) showed that increased RVent values in case of ANTs were mainly located at the lung boundary within the lung ROI. Similarly, M-REG resulted in increased JVent values at the boundary. In concordance with the previously described observations, the regional analysis (see Figure 4) shows a clear gradient in the JVent results for all registration results besides REF, manifesting as a negative (underestimation) relative difference for upper quadrants (e.g., −86% for ANTs UR) and positive (overestimation) for lower quadrants (e.g., 36% for F-REG LR). Parameters RVent and QA mainly slightly underestimated the functional parameters (e.g., −11% for F-REG UR and − 2% for QA LR), with the exception of the ANTs mean values results, which showed high overestimation for lower quadrants (e.g., 116% for LR). This specific pattern disappeared for median values, while all other patterns prevailed. See Table 2 for the summary of all results.

**Table 2:** Relative differences in relation to the predefined functional values for all registration variants depending on quadrant using mean (A) and median (B) values.

(A) Relative difference (mean values)												
	JVent				RVent				QA			
**Description**	**REF**	**ANTs**	**F-REG**	**M-REG**	**REF**	**ANTs**	**F-REG**	**M-REG**	**REF**	**ANTs**	**F-REG**	**M-REG**
UR	−0.03 (−0.03 to −0.03)	−0.86 (−0.88 to −0.73)	−0.50 (−0.55 to −0.31)	−0.61 (−1.19 to −0.30)	−0.03 (−0.04 to −0.03)	0.05 (0.02 to 0.15)	−0.11 (−0.12 to −0.09)	−0.09 (−0.13 to −0.06)	−0.00 (−0.01 to 0.00)	−0.00 (−0.01 to 0.00)	−0.19 (−0.21 to −0.17)	−0.09 (−0.14 to −0.06)
UL	−0.03 (−0.03 to −0.03)	−0.85 (−0.88 to −0.77)	−0.51 (−0.55 to −0.33)	−0.66 (−0.97 to −0.32)	−0.03 (−0.04 to −0.03)	0.05 (0.02 to 0.11)	−0.10 (−0.11 to −0.08)	−0.10 (−0.12 to −0.07)	−0.00 (−0.01 to 0.00)	−0.01 (−0.02 to 0.00)	−0.19 (−0.20 to −0.19)	−0.09 (−0.14 to −0.07)
LR	−0.03 (−0.03 to −0.03)	0.69 (0.60 to 0.73)	0.36 (0.25 to 0.45)	0.03 (−0.10 to 0.31)	−0.03 (−0.04 to −0.03)	1.16 (0.73 to 2.03)	−0.07 (−0.08 to −0.05)	−0.05 (−0.07 to −0.03)	0.00 (−0.00 to 0.00)	−0.02 (−0.04 to −0.02)	−0.17 (−0.17 to −0.16)	−0.10 (−0.14 to −0.06)
LL	−0.03 (−0.03 to −0.03)	0.69 (0.59 to 0.74)	0.40 (0.26 to 0.46)	−0.18 (−0.50 to 0.22)	−0.04 (−0.04 to −0.03)	0.63 (0.49 to 0.96)	−0.08 (−0.09 to −0.06)	−0.06 (−0.09 to −0.04)	−0.00 (−0.01 to 0.00)	−0.02 (−0.04 to −0.01)	−0.17 (−0.19 to −0.15)	−0.13 (−0.18 to −0.07)
Omnibus quadrants	3.27e−09 (****)	1.84e−10 (****)	2.07e−10 (****)	1.49e−05 (****)	9.36e−01 (n.s.)	2.77e−10 (****)	4.85e−05 (****)	3.04e−04 (***)	6.02e−01 (n.s.)	6.02e−04 (***)	2.71e−04 (***)	6.82e−01 (n.s.)
UR vs. LR	1.01e−04 (***)	8.86e−05 (****)	8.86e−05 (****)	1.40e−04 (***)	− (−)	8.86e−05 (****)	5.93e−04 (***)	5.11e−03 (**)	− (−)	2.50e−03 (**)	1.11e−02 (*)	− (−)
UL vs. LL	3.81e−05 (****)	8.86e−05 (****)	8.86e−05 (****)	1.02e−03 (**)	− (−)	8.86e−05 (****)	3.19e−03 (**)	5.73e−03 (**)	− (−)	7.19e−03 (**)	4.49e−04 (***)	− (−)
**Description**	**Omnibus REF**	**JVent vs. Rvent**	**JVent vs. QA**	**RVent vs. QA**	**Omnibus F-REG**	**JVent vs. Rvent**	**JVent vs. QA**	**RVent vs. QA**				
UR	1.95e−07 (****)	4.79e−02 (*)	8.86e−05 (****)	8.86e−05 (****)	2.06e−09 (****)	8.86e−05 (****)	8.86e−05 (****)	8.86e−05 (****)				
UL	1.37e−07 (****)	3.19e−03 (**)	8.86e−05 (****)	8.86e−05 (****)	1.25e−08 (****)	8.86e−05 (****)	1.40e−04 (***)	8.86e−05 (****)				
LR	8.76e−08 (****)	1.11e−02 (*)	8.86e−05 (****)	8.86e−05 (****)	2.06e−09 (****)	8.86e−05 (****)	8.86e−05 (****)	8.86e−05 (****)				
LL	5.06e−08 (****)	6.42e−03 (**)	8.86e−05 (****)	8.86e−05 (****)	2.06e−09 (****)	8.86e−05 (****)	8.86e−05 (****)	8.86e−05 (****)				
**Description**	**Omnibus ANTs**	**JVent vs. Rvent**	**JVent vs. QA**	**RVent vs. QA**	**Omnibus M-REG**	**JVent vs. Rvent**	**JVent vs. QA**	**RVent vs. QA**				
UR	5.06e−08 (****)	8.86e−05 (****)	8.86e−05 (****)	8.92e−04 (***)	5.03e−06 (****)	1.03e−04 (***)	1.40e−04 (***)	7.94e−01 (n.s.)				
UL	1.25e−08 (****)	8.86e−05 (****)	8.86e−05 (****)	1.89e−04 (***)	5.29e−06 (****)	1.03e−04 (***)	1.03e−04 (***)	7.65e−01 (n.s.)				
LR	8.76e−08 (****)	1.94e−03 (**)	8.86e−05 (****)	8.86e−05 (****)	9.59e−04 (***)	4.79e−02 (*)	1.00e−02 (**)	2.19e−04 (***)				
LL	2.91e−07 (****)	2.96e−01 (n.s.)	8.86e−05 (****)	8.86e−05 (****)	2.12e−01 (n.s.)	− (−)	− (−)	− (−)				

(B) Relative difference (median values)												
	JVent				RVent				QA			
**Description**	**REF**	**ANTs**	**F-REG**	**M-REG**	**REF**	**ANTs**	**F-REG**	**M-REG**	**REF**	**ANTs**	**F-REG**	**M-REG**
UR	−0.03 (−0.03 to −0.03)	−0.93 (−0.94 to −0.85)	−0.56 (−0.58 to −0.33)	−1.01 (−1.64 to −0.65)	−0.03 (−0.04 to −0.03)	−0.03 (−0.04 to −0.02)	−0.06 (−0.06 to −0.05)	−0.05 (−0.09 to −0.03)	0.00 (−0.01 to 0.01)	−0.01 (−0.01 to 0.00)	−0.03 (−0.04 to −0.03)	−0.09 (−0.10 to −0.06)
UL	−0.03 (−0.03 to −0.03)	−0.92 (−0.94 to −0.84)	−0.54 (−0.59 to −0.37)	−1.04 (−1.36 to −0.69)	−0.03 (−0.04 to −0.03)	−0.02 (−0.03 to −0.02)	−0.06 (−0.06 to −0.05)	−0.05 (−0.07 to −0.04)	−0.00 (−0.00 to 0.01)	−0.01 (−0.01 to 0.00)	−0.03 (−0.04 to −0.03)	−0.07 (−0.09 to −0.06)
LR	−0.03 (−0.03 to −0.03)	0.70 (0.63 to 0.75)	0.43 (0.36 to 0.54)	−0.36 (−0.57 to −0.13)	−0.03 (−0.05 to −0.03)	−0.02 (−0.03 to −0.01)	−0.04 (−0.05 to −0.03)	−0.04 (−0.05 to −0.01)	−0.00 (−0.01 to 0.01)	−0.01 (−0.02 to −0.01)	−0.04 (−0.04 to −0.03)	−0.08 (−0.10 to −0.06)
LL	−0.03 (−0.03 to −0.03)	0.69 (0.62 to 0.74)	0.47 (0.34 to 0.55)	−0.56 (−0.80 to −0.17)	−0.03 (−0.04 to −0.03)	−0.03 (−0.04 to −0.02)	−0.05 (−0.06 to −0.04)	−0.06 (−0.06 to −0.04)	−0.00 (−0.01 to 0.00)	−0.01 (−0.02 to −0.00)	−0.04 (−0.04 to −0.02)	−0.09 (−0.10 to −0.07)
Omnibus quadrants	1.00e+00 (n.s.)	2.01e−10 (****)	2.13e−10 (****)	1.62e−04 (***)	7.96e−01 (n.s.)	7.13e−02 (n.s.)	2.21e−03 (**)	1.92e−01 (n.s.)	5.16e−01 (n.s.)	1.48e−02 (*)	6.02e−01 (n.s.)	7.39e−01 (n.s.)
UR vs. LR	− (−)	8.86e−05 (****)	8.86e−05 (****)	2.93e−04 (***)	− (−)	− (−)	6.81e−04 (***)	− (−)	− (−)	3.33e−02 (*)	− (−)	− (−)
UL vs. LL	− (−)	8.86e−05 (****)	8.86e−05 (****)	3.59e−03 (**)	− (−)	− (−)	8.97e−03 (**)	− (−)	− (−)	2.51e−02 (*)	− (−)	− (−)
**Description**	**Omnibus REF**	**JVent vs. Rvent**	**JVent vs. QA**	**RVent vs. QA**	**Omnibus F-REG**	**JVent vs. Rvent**	**JVent vs. QA**	**RVent vs. QA**				
UR	1.95e−07 (****)	4.38e−02 (*)	8.86e−05 (****)	8.86e−05 (****)	5.33e−09 (****)	8.86e−05 (****)	8.86e−05 (****)	1.63e−04 (***)				
UL	8.76e−08 (****)	2.51e−02 (*)	8.86e−05 (****)	8.86e−05 (****)	5.33e−09 (****)	8.86e−05 (****)	8.86e−05 (****)	1.03e−04 (***)				
LR	1.37e−07 (****)	3.04e−02 (*)	8.86e−05 (****)	8.86e−05 (****)	2.50e−07 (****)	8.86e−05 (****)	8.86e−05 (****)	2.28e−02 (*)				
LL	8.76e−08 (****)	5.11e−03 (**)	8.86e−05 (****)	8.86e−05 (****)	5.06e−08 (****)	8.86e−05 (****)	8.86e−05 (****)	1.51e−03 (**)				
**Description**	**Omnibus ANTs**	**JVent vs. Rvent**	**JVent vs. QA**	**RVent vs. QA**	**Omnibus M-REG**	**JVent vs. Rvent**	**JVent vs. QA**	**RVent vs. QA**				
UR	2.64e−08 (****)	8.86e−05 (****)	8.86e−05 (****)	7.80e−04 (***)	2.91e−07 (****)	8.86e−05 (****)	1.03e−04 (***)	5.11e−03 (**)				
UL	2.06e−09 (****)	8.86e−05 (****)	8.86e−05 (****)	8.86e−05 (****)	1.25e−08 (****)	8.86e−05 (****)	8.86e−05 (****)	1.02e−03 (**)				
LR	1.95e−07 (****)	8.86e−05 (****)	8.86e−05 (****)	5.22e−02 (n.s.)	1.18e−06 (****)	1.89e−04 (***)	3.90e−04 (***)	2.19e−04 (***)				
LL	1.25e−08 (****)	8.86e−05 (****)	8.86e−05 (****)	2.50e−03 (**)	3.90e−04 (***)	1.16e−03 (**)	1.71e−03 (**)	1.32e−03 (**)				

Respective omnibus and *post-hoc* test results for differences regarding quadrants (omnibus quadrants) and parameters (omnibus registration abbreviation) are included as well. *p* ≤ 0.05 (*), *p* ≤ 0.01 (**), *p* ≤ 0.001 (***), *p* ≤ 0.0001 (****). Please note that the numbers displayed in this table are rounded to two decimal places. As a result, different values may appear identical despite minor differences in their actual values. REF: reference (known) registration, ANTs: advanced normalization tools, F-Reg: Forsberg registration, M-Reg: Matlab registration, RVent: regional ventilation, JVent: Jacobian determinant ventilation, QA: perfusion amplitude, UR: upper right, UL: upper left, LR: lower right, LL: lower left.

#### 4.1.3 Sensitivity and specificity

**Figure 5:**
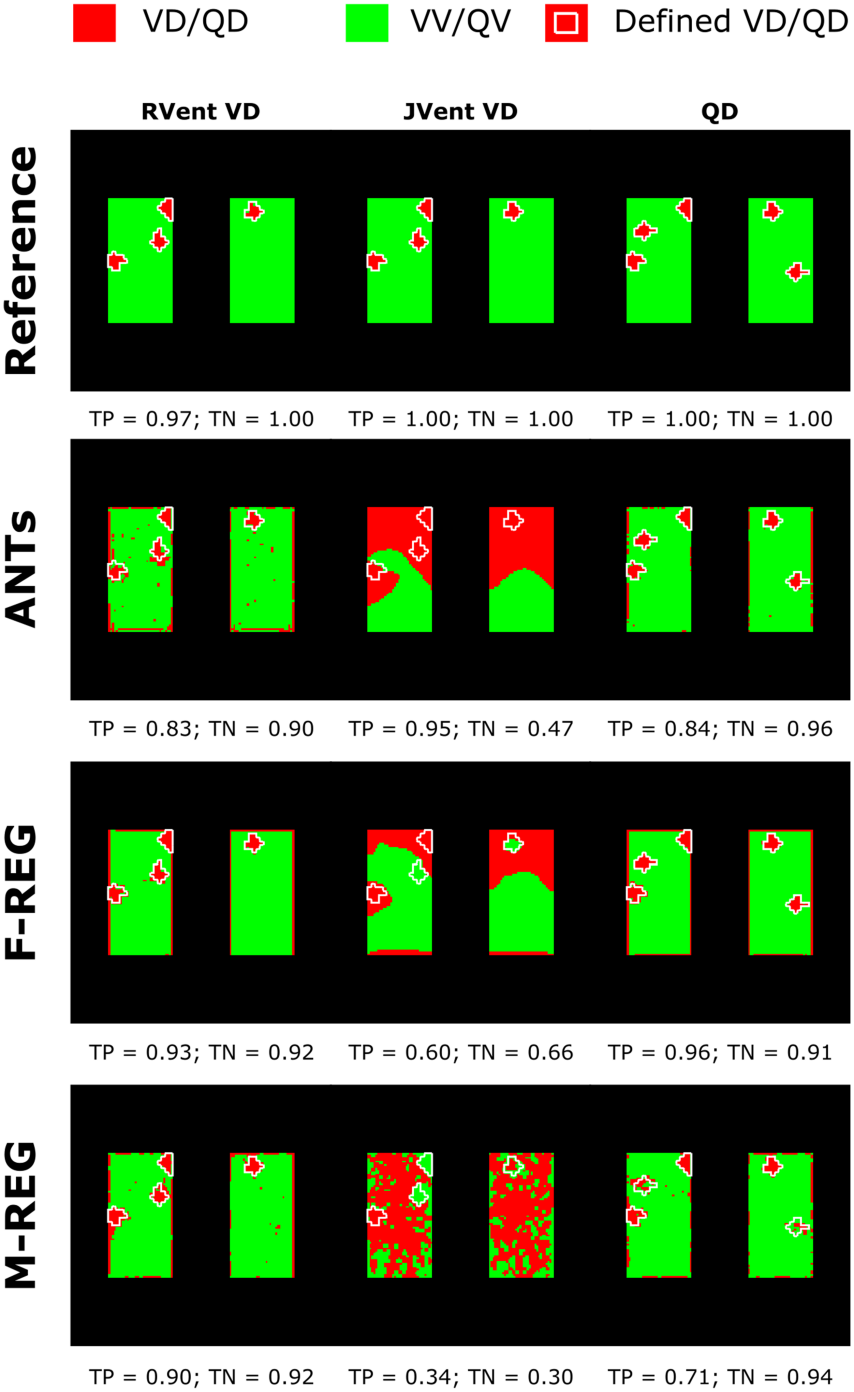
Regional concordance of the ventilation defects (VD) derived from RVent, JVent, and the perfusion defects (QD) derived from QA using registrations with Reference, ANTs, F-REG, and M-REG. As expected, the reference registration results in nearly perfect correspondence to the defined classes. VD derived from JVent results in overestimation, especially at the upper regions, leading to low specificity (true negative rate) scores. All VD/QD measurements show problems near the edges. REF: reference (known) registration, ANTs: advanced normalization tools, F-Reg: Forsberg registration, M-Reg: Matlab registration, RVent: regional ventilation, JVent: Jacobian determinant ventilation, QA: perfusion amplitude, TP: true positive rate, TN: true negative rate.

Assessments of regional defect maps, as demonstrated in Figure 5, JVent showed a higher amount of VD in the upper lung regions, which led to a high true positive and low true negative rate in this case. Remarkably, JVent was able to identify the VD|QD_High_ defect class, although it was located in the lower half region with higher sensitivity. All registration variants, except for REF, had a noticeable amount of VD/QD at the lung boundary. M-REG resulted in unusable JVent. In general, RVent and QA identified the defined VD and QD regions.

**Figure 6:**
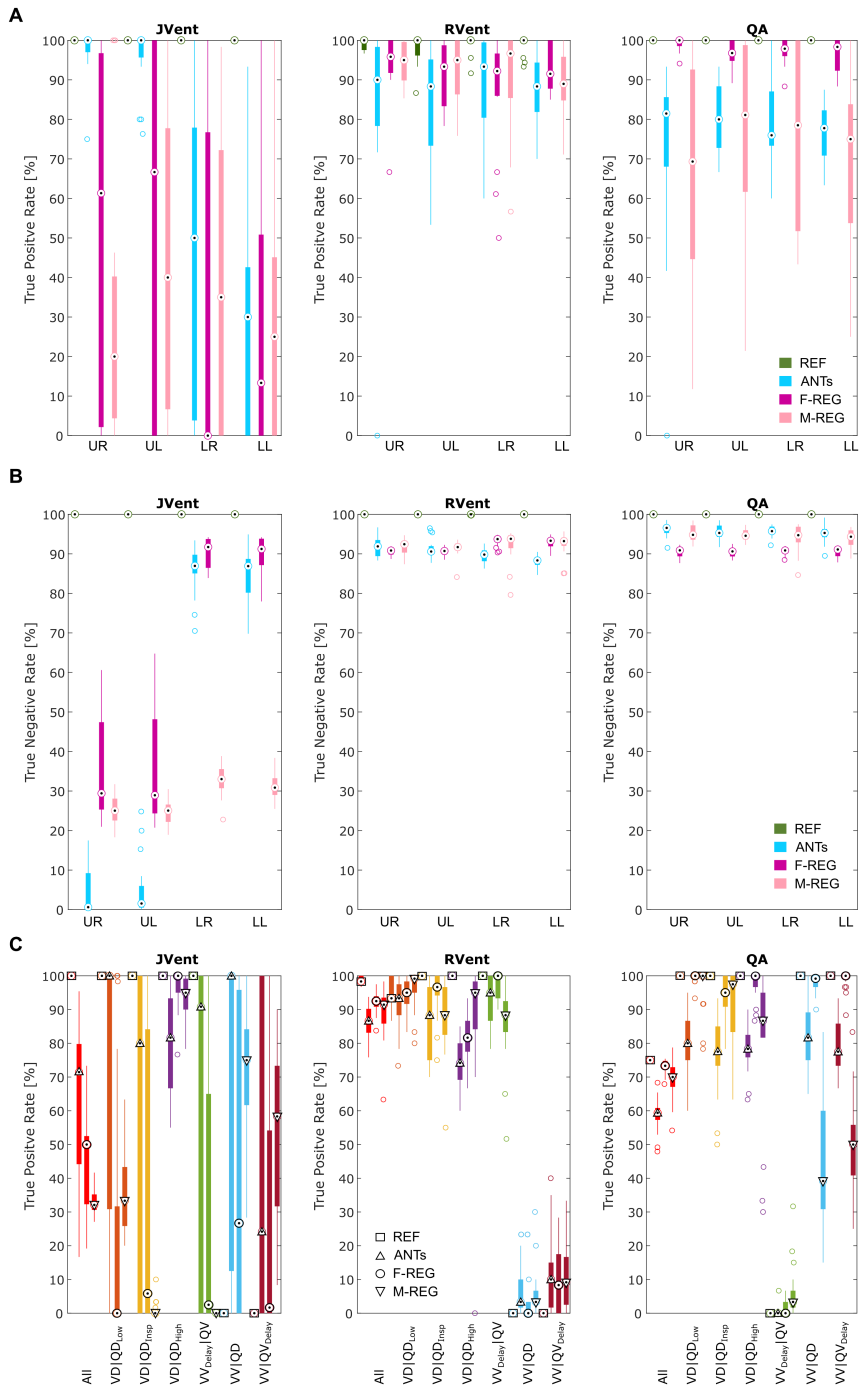
True positive **(A)** and true negative **(B)** rates for VD and QD based on thresholded JVent, RVent, and QA parameters according to the 90th percentile × 0.4 with different registration methods. Please note the considerably lower rates of JVent in comparison to the signal-based RVent and QA. **(C)** Shows the performance for the whole lung ROI depending on the defect class and registration algorithm (as indicated by different symbols at the median position). REF: reference (known) registration, ANTs: advanced normalization tools, F-Reg: Forsberg registration, M-Reg: Matlab registration, RVent: regional ventilation, JVent: Jacobian determinant ventilation, QA: perfusion amplitude, All: all defect classes, VD|QD_Low_: ventilation and perfusion defect below inspiration signal level, VD|QD_Insp_: ventilation and perfusion defect at inspiration level, VD|QD_High_: ventilation and perfusion defect at high signal level, VV_Delay_|QV: ventilated and perfused volume with delayed ventilation, VV|QD: ventilated volume with perfusion defect, VV|QV_Delay_: ventilated and perfused volume with delayed perfusion, UR: upper right, UL: upper left, LR: lower right, LL: lower left.

In concordance with the presented example, as summarized in Table 3 and Figure 6A, the sensitivity was very low for JVent (0–16%) for the non-REF registration variants in the lower quadrants in comparison to upper quadrants (29–99%, significant for ANTs, *p* ≤ 0.01). Contrary to this, RVent and QA displayed overall high sensitivity (68–100%) across all quadrants and registration variants. Differences regarding quadrants were only found for QA with F-REG between UR and LR (100% vs. 97%, *p* ≤ 0.05).

**Table 3:** True positive (A) and true negative (B) results based on VD calculated from JVent, RVent, and perfusion defect (QD) derived from QA.

(A) True positive rate												
	JVent				RVent				QA			
**Description**	**REF**	**ANTs**	**F-REG**	**M-REG**	**REF**	**ANTs**	**F-REG**	**M-REG**	**REF**	**ANTs**	**F-REG**	**M-REG**
UR	1.00 (0.50–1.00)	0.99 (0.38–1.00)	0.29 (0.00–0.86)	0.16 (0.00–0.21)	1.00 (0.48–1.00)	0.82 (0.00–0.95)	0.92 (0.33–1.00)	0.93 (0.43–0.97)	1.00 (1.00–1.00)	0.79 (0.67–0.85)	1.00 (0.98–1.00)	0.68 (0.42–0.92)
UL	1.00 (1.00–1.00)	0.99 (0.80–1.00)	0.50 (0.00–0.97)	0.30 (0.00–0.69)	1.00 (0.93–1.00)	0.84 (0.65–0.93)	0.92 (0.81–0.98)	0.94 (0.82–1.00)	1.00 (1.00–1.00)	0.78 (0.72–0.86)	0.97 (0.93–1.00)	0.73 (0.44–0.96)
LR	1.00 (0.50–1.00)	0.16 (0.00–0.62)	0.00 (0.00–0.48)	0.00 (0.00–0.51)	1.00 (0.46–1.00)	0.83 (0.30–0.97)	0.87 (0.25–0.97)	0.94 (0.28–0.99)	1.00 (1.00–1.00)	0.76 (0.72–0.84)	0.97 (0.94–1.00)	0.70 (0.51–1.00)
LL	1.00 (1.00–1.00)	0.06 (0.00–0.42)	0.00 (0.00–0.50)	0.16 (0.00–0.44)	1.00 (0.95–1.00)	0.84 (0.76–0.94)	0.90 (0.87–1.00)	0.87 (0.83–0.95)	1.00 (1.00–1.00)	0.78 (0.70–0.82)	0.98 (0.92–1.00)	0.74 (0.45–0.84)
Omnibus quadrants	7.53e−01 (n.s.)	5.21e−05 (****)	5.02e−01 (n.s.)	7.23e−01 (n.s.)	8.98e−01 (n.s.)	9.77e−01 (n.s.)	6.14e−01 (n.s.)	6.73e−01 (n.s.)	6.66e−01 (n.s.)	9.78e−01 (n.s.)	4.21e−02 (*)	7.51e−01 (n.s.)
UR vs. LR	− (−)	3.55e−03 (**)	− (−)	− (−)	− (−)	− (−)	− (−)	− (−)	− (−)	− (−)	3.98e−02 (*)	− (−)
UL vs. LL	− (−)	1.23e−03 (**)	− (−)	− (−)	− (−)	− (−)	− (−)	− (−)	− (−)	− (−)	6.58e−01 (n.s.)	− (−)
**Description**	**Omnibus REF**	**JVent vs. RVent**	**JVent vs. QA**	**RVent vs. QA**	**Omnibus F-REG**	**JVent vs. Rvent**	**JVent vs. QA**	**RVent vs. QA**				
UR	8.23e−03 (**)	1.25e−01 (n.s.)	2.19e−01 (n.s.)	3.91e−02 (*)	4.61e−05 (****)	1.02e−02 (*)	2.00e−03 (**)	4.03e−03 (**)				
UL	6.22e−02 (n.s.)	− (−)	− (−)	− (−)	9.36e−03 (**)	1.61e−02 (*)	1.30e−02 (*)	1.02e−01 (n.s.)				
LR	6.63e−02 (n.s.)	− (−)	− (−)	− (−)	9.93e−04 (***)	3.23e−03 (**)	4.84e−04 (***)	1.33e−02 (*)				
LL	2.24e−02 (*)	2.50e−01 (n.s.)	5.00e−01 (n.s.)	6.25e−02 (n.s.)	3.65e−06 (****)	3.29e−04 (***)	1.52e−04 (***)	1.51e−02 (*)				
**Description**	**Omnibus ANTs**	**JVent vs. RVent**	**JVent vs. QA**	**RVent vs. QA**	**Omnibus M-REG**	**JVent vs. Rvent**	**JVent vs. QA**	**RVent vs. QA**				
UR	8.68e−03 (**)	7.32e−04 (***)	3.90e−01 (n.s.)	6.47e−01 (n.s.)	2.15e−03 (**)	4.88e−04 (***)	1.48e−03 (**)	4.21e−01 (n.s.)				
UL	2.01e−03 (**)	3.66e−04 (***)	1.41e−02 (*)	8.11e−01 (n.s.)	8.37e−04 (***)	7.10e−04 (***)	1.48e−02 (*)	8.40e−02 (n.s.)				
LR	2.55e−03 (**)	8.54e−04 (***)	2.47e−03 (**)	8.11e−01 (n.s.)	2.67e−03 (**)	1.22e−03 (**)	1.17e−03 (**)	8.87e−01 (n.s.)				
LL	2.06e−04 (***)	5.01e−04 (***)	3.41e−04 (***)	4.86e−02 (*)	1.92e−05 (****)	3.50e−04 (***)	1.96e−04 (***)	1.22e−01 (n.s.)				

(B) True negative rate												
	JVent				RVent				QA			
**Description**	**REF**	**ANTs**	**F-REG**	**M-REG**	**REF**	**ANTs**	**F-REG**	**M-REG**	**REF**	**ANTs**	**F-REG**	**M-REG**
UR	1.00 (0.50–1.00)	0.01 (0.00–0.09)	0.29 (0.25–0.47)	0.25 (0.23–0.28)	1.00 (0.50–1.00)	0.92 (0.89–0.93)	0.91 (0.90–0.91)	0.92 (0.90–0.93)	1.00 (1.00–1.00)	0.97 (0.95–0.97)	0.91 (0.89–0.92)	0.95 (0.94–0.97)
UL	1.00 (1.00–1.00)	0.02 (0.01–0.06)	0.29 (0.24–0.48)	0.25 (0.22–0.27)	1.00 (1.00–1.00)	0.91 (0.90–0.92)	0.91 (0.90–0.91)	0.92 (0.91–0.93)	1.00 (1.00–1.00)	0.95 (0.94–0.97)	0.91 (0.89–0.91)	0.95 (0.94–0.96)
LR	1.00 (0.50–1.00)	0.87 (0.85–0.90)	0.92 (0.86–0.94)	0.33 (0.31–0.36)	1.00 (1.00–1.00)	0.90 (0.88–0.91)	0.94 (0.93–0.94)	0.94 (0.91–0.95)	1.00 (1.00–1.00)	0.96 (0.95–0.97)	0.91 (0.90–0.91)	0.95 (0.93–0.97)
LL	1.00 (1.00–1.00)	0.87 (0.80–0.89)	0.91 (0.87–0.94)	0.31 (0.29–0.33)	1.00 (1.00–1.00)	0.88 (0.87–0.89)	0.93 (0.92–0.94)	0.93 (0.92–0.94)	1.00 (1.00–1.00)	0.95 (0.94–0.96)	0.91 (0.89–0.91)	0.94 (0.92–0.96)
Omnibus quadrants	7.53e−01 (n.s.)	1.45e−10 (****)	1.84e−10 (****)	6.67e−07 (****)	7.48e−01 (n.s.)	2.89e−05 (****)	1.23e−07 (****)	4.06e−02 (*)	6.66e−01 (n.s.)	4.40e−01 (n.s.)	6.50e−01 (n.s.)	9.32e−01 (n.s.)
UR vs. LR	− (−)	8.84e−05 (****)	8.86e−05 (****)	1.40e−04 (***)	− (−)	4.49e−04 (***)	1.63e−04 (***)	1.56e−01 (n.s.)	− (−)	− (−)	− (−)	− (−)
UL vs. LL	− (−)	8.86e−05 (****)	8.86e−05 (****)	2.19e−04 (***)	− (−)	2.93e−04 (***)	3.38e−04 (***)	4.38e−02 (*)	− (−)	− (−)	− (−)	− (−)
**Description**	**Omnibus REF**	**JVent vs. RVent**	**JVent vs. QA**	**RVent vs. QA**	**Omnibus F-REG**	**JVent vs. RVent**	**JVent vs. QA**	**RVent vs. QA**				
UR	6.95e−02 (n.s.)	− (−)	− (−)	− (−)	2.50e−07 (****)	8.86e−05 (****)	8.86e−05 (****)	7.94e−01 (n.s.)				
UL	1.00e+00 (n.s.)	− (−)	− (−)	− (−)	1.36e−07 (****)	8.86e−05 (****)	8.86e−05 (****)	3.34e−01 (n.s.)				
LR	1.74e−01 (n.s.)	− (−)	− (−)	− (−)	1.67e−04 (***)	2.90e−03 (**)	5.75e−01 (n.s.)	8.86e−05 (****)				
LL	1.35e−01 (n.s.)	− (−)	− (−)	− (−)	1.29e−03 (**)	8.96e−03 (**)	9.70e−01 (n.s.)	2.19e−04 (***)				
**Description**	**Omnibus ANTs**	**JVent vs. RVent**	**JVent vs. QA**	**RVent vs. QA**	**Omnibus M-REG**	**JVent vs. RVent**	**JVent vs. QA**	**RVent vs. QA**				
UR	2.06e−09 (****)	8.84e−05 (****)	8.86e−05 (****)	8.86e−05 (****)	5.33e−09 (****)	8.86e−05 (****)	8.86e−05 (****)	1.20e−04 (***)				
UL	2.06e−09 (****)	8.86e−05 (****)	8.86e−05 (****)	8.86e−05 (****)	2.06e−09 (****)	8.86e−05 (****)	8.86e−05 (****)	8.86e−05 (****)				
LR	9.66e−07 (****)	2.51e−02 (*)	1.03e−04 (***)	8.86e−05 (****)	5.33e−09 (****)	8.86e−05 (****)	8.86e−05 (****)	1.63e−04 (***)				
LL	1.37e−07 (****)	9.46e−03 (**)	8.86e−05 (****)	8.86e−05 (****)	5.06e−08 (****)	8.86e−05 (****)	8.86e−05 (****)	5.73e−03 (**)				

Respective omnibus and post-hoc test results for differences regarding quadrants (omnibus quadrants) and parameters (omnibus registration abbreviation) are included as well. *p* ≤ 0.05 (*), *p* ≤ 0.01 (**), *p* ≤ 0.001 (***), *p* ≤ 0.0001 (****). Please note that the numbers displayed in this table are rounded to two decimal places. As a result, different values may appear identical despite minor differences in their actual values. REF: reference (known) registration, ANTs: advanced normalization tools, F-Reg: Forsberg registration, M-Reg: Matlab registration, RVent: regional ventilation, JVent: Jacobian determinant ventilation, QD: perfusion defect, UR: upper right, UL: upper left, LR: lower right, LL: lower left.

The specificity analysis (see Figure 6B) showed an inverted performance for JVent in comparison with previously described sensitivity results: 87% for LR/LL vs. 1 and 2% for UR/UL (ANTs). As before, this pattern was less pronounced but significant for F-REG and even more so for M-REG. The specificity for RVent and QA was very high (88–97%) across all registration variants and quadrants.

Sensitivity results as a function of individual defect classes (see Figure 6C) confirmed the better performance of JVent for VD|QD_High_ in comparison to all other classes regarding median values and amount of dispersion. As expected, VV classes were nearly zero for RVent and QV classes were nearly zero for QA. Otherwise, the differences in class performance were less pronounced and less dispersed for RVent and QA in comparison to JVent.

### 4.2 Patient cohort

Except for ANTs (0.16 vs. 0.17, *p* = 0.15), JVent was significantly higher than the additionally filtered RVent* (0.22 vs. 0.13 (F-REG) and 0.08 vs. 0.04 (M-REG), *p* ≤ 0.01). All registration variants resulted in significantly different values. See Table 4 for a summary of all results.

**Table 4:** RVent and JVent median (interquartile) values of COPD patient cohort (*n* = 36).

Description	RVent*	JVent	RVent* vs. JVent
ANTs	0.17 (0.11–0.22)	0.16 (0.12–0.21)	1.47e−01 (n.s.)
F-REG	0.13 (0.08–0.18)	0.22 (0.16–0.32)	7.39e−27 (****)
M-REG	0.04 (0.02–0.06)	0.08 (0.02–0.15)	4.16e−09 (****)
Omnibus	1.13e−65 (****)	5.36e−44 (****)	—
ANTs vs. F-REG	7.39e−27 (****)	7.84e−27 (****)	—
ANTs vs. M-REG	5.37e−27 (****)	2.14e−13 (****)	—
F-REG vs. M-REG	8.00e−27 (****)	2.21e−25 (****)	—

Except for ANTs, all values were significantly different in regard to method (RVent vs. JVent) and registration algorithm. *p* ≤ 0.05 (*), *p* ≤ 0.01 (**), *p* ≤ 0.001 (***), *p* ≤ 0.0001 (****). Please note that the numbers displayed in this table are rounded to two decimal places. As a result, different values may appear identical despite minor differences in their actual values. ANTs: advanced normalization tools, F-Reg: Forsberg registration, M-Reg: Matlab registration, RVent*: filtered regional ventilation, JVent: Jacobian determinant ventilation.

The quadrant analysis (see Table 5) showed significantly increased JVent-derived VD in comparison to RVent and CT for F-REG and especially ANTs: 54/60% UR/UL vs. 16/10% LR/LL (ANTs), 47/58% UR/UL vs. 27/25% LR/LL (F-REG). Contrary to this, RVent* showed a reversed defect ratio: 14/13% UR/UL vs. 38/42% LR/LL (ANTs) and 20/20% UR/UL vs. 44/46% LR/LL (F-REG). This defect distribution was also more similar to CT: 0.23/0.14 UR/UL vs. 42/37% LR/LL. M-REG demonstrated overall increased defect percentages and less pronounced differences between upper and lower lungs. These results were also reflected in the CT overlap coefficient (see Table 6), as UR and UL showed significantly higher coefficients for RVent* in comparison with JVent: 0.72/0.73 vs. 0.62/0.53 (ANTs), 0.68/0.70 vs. 0.57/0.51 (F-REG), and 0.46/0.43 vs. 0.37/0.32, *p* ≤ 0.001.

**Table 5:** Overview of ventilation defects (VD) distribution for different quadrants, methods, and registration algorithms.

	ANTs						
	CT	JVent	RVent*	Omnibus ANTS	CT vs. JVent	CT vs. RVent*	JVent vs. RVent*
UR	0.23 (0.02–0.50)	0.54 (0.23–0.89)	0.14 (0.01–0.41)	4.47e−27 (****)	1.87e−12 (****)	9.53e−05 (****)	2.98e−22 (****)
UL	0.14 (0.02–0.44)	0.60 (0.20–0.96)	0.13 (0.01–0.35)	5.44e−28 (****)	3.36e−16 (****)	1.28e−02 (*)	1.72e−23 (****)
LR	0.42 (0.11–0.65)	0.16 (0.04–0.36)	0.38 (0.12–0.60)	9.86e−13 (****)	6.50e−12 (****)	2.85e−01 (n.s.)	1.52e−15 (****)
LL	0.37 (0.08–0.56)	0.10 (0.02–0.32)	0.42 (0.12–0.60)	1.15e−15 (****)	1.45e−11 (****)	2.58e−01 (n.s.)	2.37e−17 (****)
Omnibus quadrants	3.28e−13 (****)	3.86e−45 (****)	7.27e−22 (****)				
UR vs. LR	1.51e−05 (****)	6.92e−22 (****)	3.12e−11 (****)				
UL vs. LL	6.77e−08 (****)	1.98e−23 (****)	6.87e−14 (****)				

	F-REG						
	CT	JVent	RVent*	Omnibus F-REG	CT vs. JVent	CT vs. RVent*	JVent vs. RVent*
UR	0.23 (0.02–0.50)	0.47 (0.30–0.81)	0.20 (0.08–0.43)	1.04e−23 (****)	4.13e−14 (****)	5.88e−02 (n.s.)	1.66e−23 (****)
UL	0.14 (0.02–0.44)	0.58 (0.37–0.79)	0.20 (0.07–0.35)	6.99e−33 (****)	7.15e−18 (****)	6.89e−01 (n.s.)	2.65e−25 (****)
LR	0.42 (0.11–0.65)	0.27 (0.17–0.39)	0.44 (0.19–0.66)	5.66e−06 (****)	5.87e−05 (****)	2.64e−01 (n.s.)	1.14e−09 (****)
LL	0.37 (0.08–0.56)	0.25 (0.15–0.39)	0.46 (0.21–0.66)	2.23e−07 (****)	8.82e−03 (**)	3.78e−04 (***)	3.09e−12 (****)
Omnibus quadrants	3.28e−13 (****)	6.39e−29 (****)	2.49e−17 (****)				
UR vs. LR	1.51e−05 (****)	3.61e−17 (****)	1.22e−10 (****)				
UL vs. LL	6.77e−08 (****)	1.42e−19 (****)	3.30e−14 (****)				

	M-REG						
	CT	JVent	RVent*	Omnibus M-REG	CT vs. JVent	CT vs. RVent*	JVent vs. RVent*
UR	0.23 (0.02–0.50)	0.75 (0.70–0.77)	0.65 (0.59–0.74)	1.16e−35 (****)	4.91e−24 (****)	6.57e−22 (****)	2.65e−19 (****)
UL	0.14 (0.02–0.44)	0.77 (0.72–0.80)	0.68 (0.60–0.75)	3.71e−38 (****)	1.01e−24 (****)	2.84e−23 (****)	6.14e−17 (****)
LR	0.42 (0.11–0.65)	0.70 (0.66–0.75)	0.71 (0.63–0.78)	2.50e−21 (****)	3.78e−19 (****)	5.68e−20 (****)	3.60e−01 (n.s.)
LL	0.37 (0.08–0.56)	0.73 (0.68–0.78)	0.73 (0.64–0.81)	6.11e−27 (****)	7.76e−21 (****)	1.04e−22 (****)	9.17e−01 (n.s.)
Omnibus quadrants	3.28e−13 (****)	2.99e−10 (****)	7.98e−03 (**)				
UR vs. LR	1.51e−05 (****)	6.40e−06 (****)	1.91e−02 (*)				
UL vs. LL	6.77e−08 (****)	5.66e−05 (****)	1.76e−03 (**)				

Significantly higher VD_JVent_ were present in the upper lung regions in comparison to the lower regions for ANTs and for F-REG. RVent showed a reversed relationship for ANTs and F-REG similar to CT. Respective omnibus and post-hoc test results for differences regarding quadrants (omnibus quadrants) and parameters (omnibus registration abbreviation) are included as well. *p* ≤ 0.05 (*), *p* ≤ 0.01 (**), *p* ≤ 0.001 (***), *p* ≤ 0.0001 (****). Please note that the numbers displayed in this table are rounded to two decimal places. As a result, different values may appear identical despite minor differences in their actual values. ANTs: advanced normalization tools, F-Reg: Forsberg registration, M-Reg: Matlab registration, RVent*: filtered regional ventilation, JVent: Jacobian determinant ventilation, UR: upper right, UL: upper left, LR: lower right, LL: lower left, VD: ventilation defect.

**Table 6:** Two-class overlap coefficient of ventilation defect (VD) and ventilated volume (VV) of RVent and JVent with CT as gold standard for different quadrants and registration algorithms.

	ANTs			F-REG			M-REG		
Description	RVent*	JVent	RVent* vs. JVent	RVent*	JVent	RVent* vs. JVent	RVent*	JVent	RVent* vs. JVent
UR	0.72 (0.59–0.89)	0.62 (0.44–0.77)	4.54e−07 (****)	0.68 (0.54–0.86)	0.57 (0.44–0.71)	3.21e−08 (****)	0.46 (0.39–0.54)	0.37 (0.29–0.53)	2.81e−10 (****)
UL	0.73 (0.54–0.90)	0.53 (0.34–0.77)	2.49e−10 (****)	0.70 (0.52–0.87)	0.51 (0.37–0.64)	3.36e−12 (****)	0.43 (0.32–0.53)	0.32 (0.23–0.47)	2.14e−12 (****)
LR	0.60 (0.52–0.76)	0.56 (0.42–0.76)	5.72e−04 (***)	0.59 (0.51–0.71)	0.53 (0.42–0.70)	6.04e−06 (****)	0.49 (0.41–0.60)	0.44 (0.35–0.55)	8.39e−09 (****)
LL	0.58 (0.48–0.74)	0.53 (0.41–0.83)	6.97e−02 (n.s.)	0.55 (0.46–0.68)	0.50 (0.41–0.76)	3.69e−03 (**)	0.47 (0.36–0.56)	0.42 (0.30–0.53)	4.15e−06 (****)
Omnibus quadrants	1.19e−11 (****)	1.23e−01 (n.s.)	—	1.77e−07 (****)	2.74e−02 (*)	—	4.22e−07 (****)	1.10e−16 (****)	—
UR vs. LR	9.43e−09 (****)	− (−)	—	2.26e−06 (****)	3.04e−01 (n.s.)	—	2.75e−02 (*)	4.93e−05 (****)	—
UL vs. LL	2.49e−06 (****)	− (−)	—	5.74e−06 (****)	3.44e−02 (*)	—	8.11e−03 (**)	4.75e−07 (****)	—

Especially for UR and UL, RVent showed significantly higher overlaps. *p* ≤ 0.05 (*), *p* ≤ 0.01 (**), *p* ≤ 0.001 (***), *p* ≤ 0.0001 (****). Please note that the numbers displayed in this table are rounded to two decimal places. As a result, different values may appear identical despite minor differences in their actual values. ANTs: advanced normalization tools, F-Reg: Forsberg registration, M-Reg: Matlab registration, RVent*: filtered regional ventilation, JVent: Jacobian determinant ventilation, UR: upper right, UL: upper left, LR: lower right, LL: lower left, VD: ventilation defect.

**Figure 7:**
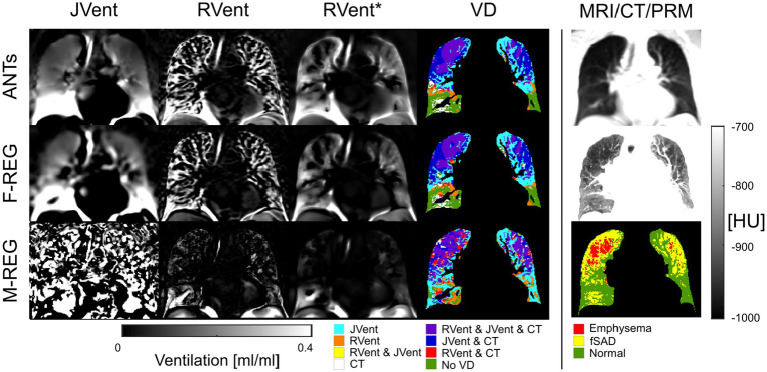
RVent without and with additional filtering (*) in the second and third columns in comparison to JVent (first column) for a male COPD (GOLD III) patient (age = 52). Rows show the three registration variants. The last column shows the expiration MR image for anatomical reference, CT in expiration, and parametric response mapping (PRM). The fourth column demonstrates the alignment of the respective ventilation defects derived from RVent* and JVent to PRM. Please note that JVent and RVent show similar VD patterns aligned with PRM located in the upper regions of the lung in this case. ANTs: advanced normalization tools, F-Reg: Forsberg registration, M-Reg: Matlab registration, RVent: regional ventilation, RVent*: filtered regional ventilation, JVent: Jacobian determinant ventilation, PRM: parametric response mapping, fSAD: functional small airway disease, VD: ventilation defect, COPD: chronic obstructive pulmonary disease.

**Figure 8:**
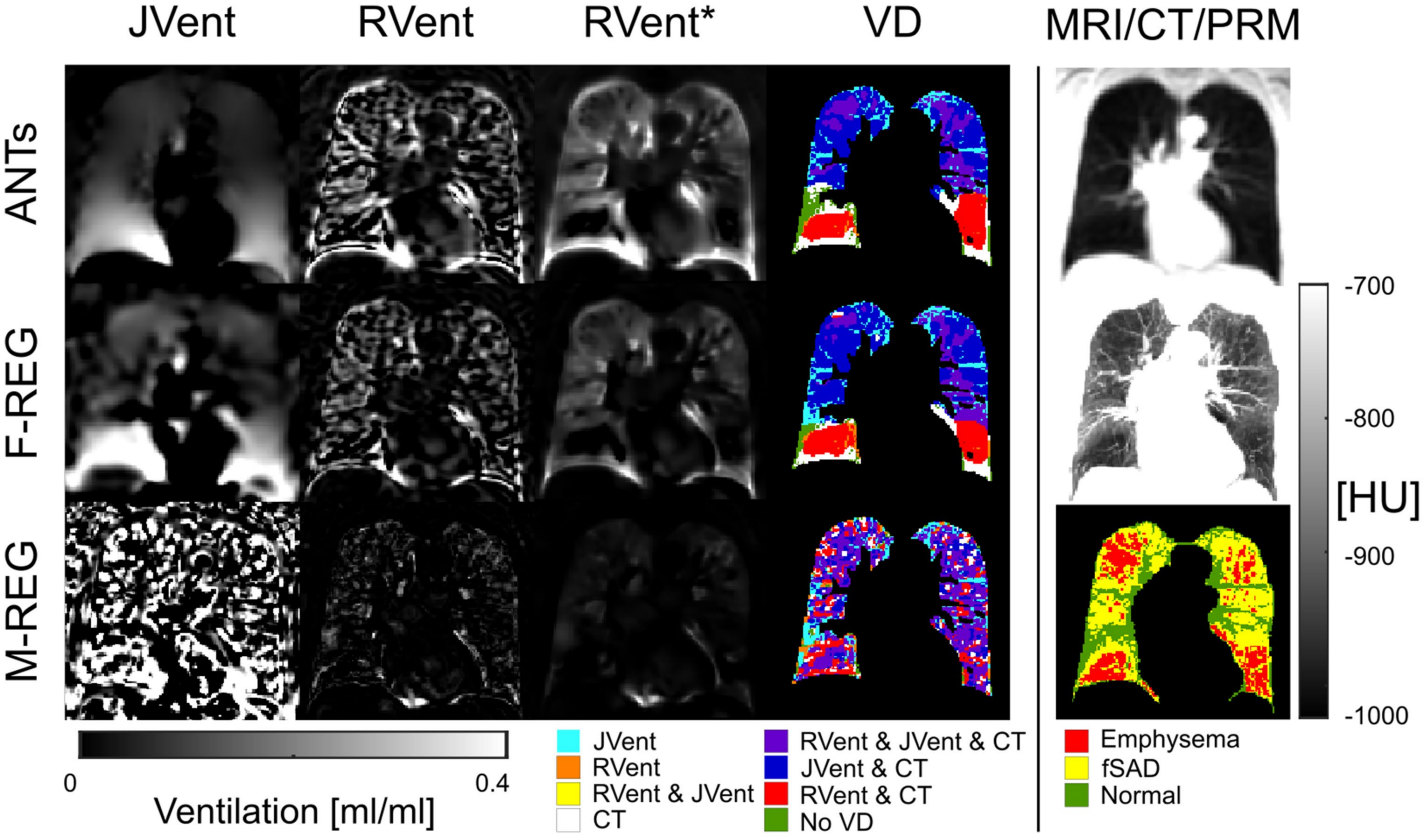
RVent without and with additional filtering (*) in the second and third columns in comparison to Jvent (first column) for a female COPD (Gold IV) patient (age = 70). Rows show the three registration variants. The last column shows the expiration MR image for anatomical reference, CT in expiration, and parametric response mapping (PRM). The fourth column demonstrates the alignment of the respective ventilation defects derived from RVent* and JVent to PRM. JVent and RVent showed PRM-aligned VD in the upper lung. RVent was also aligned in the lower parts. ANTs: advanced normalization tools, F-Reg: Forsberg registration, M-Reg: Matlab registration, RVent: regional ventilation, RVent*: filtered regional ventilation, JVent: Jacobian determinant ventilation, PRM: parametric response mapping, fSAD: functional small airway disease, VD: ventilation defect, COPD: chronic obstructive pulmonary disease.

**Figure 9:**
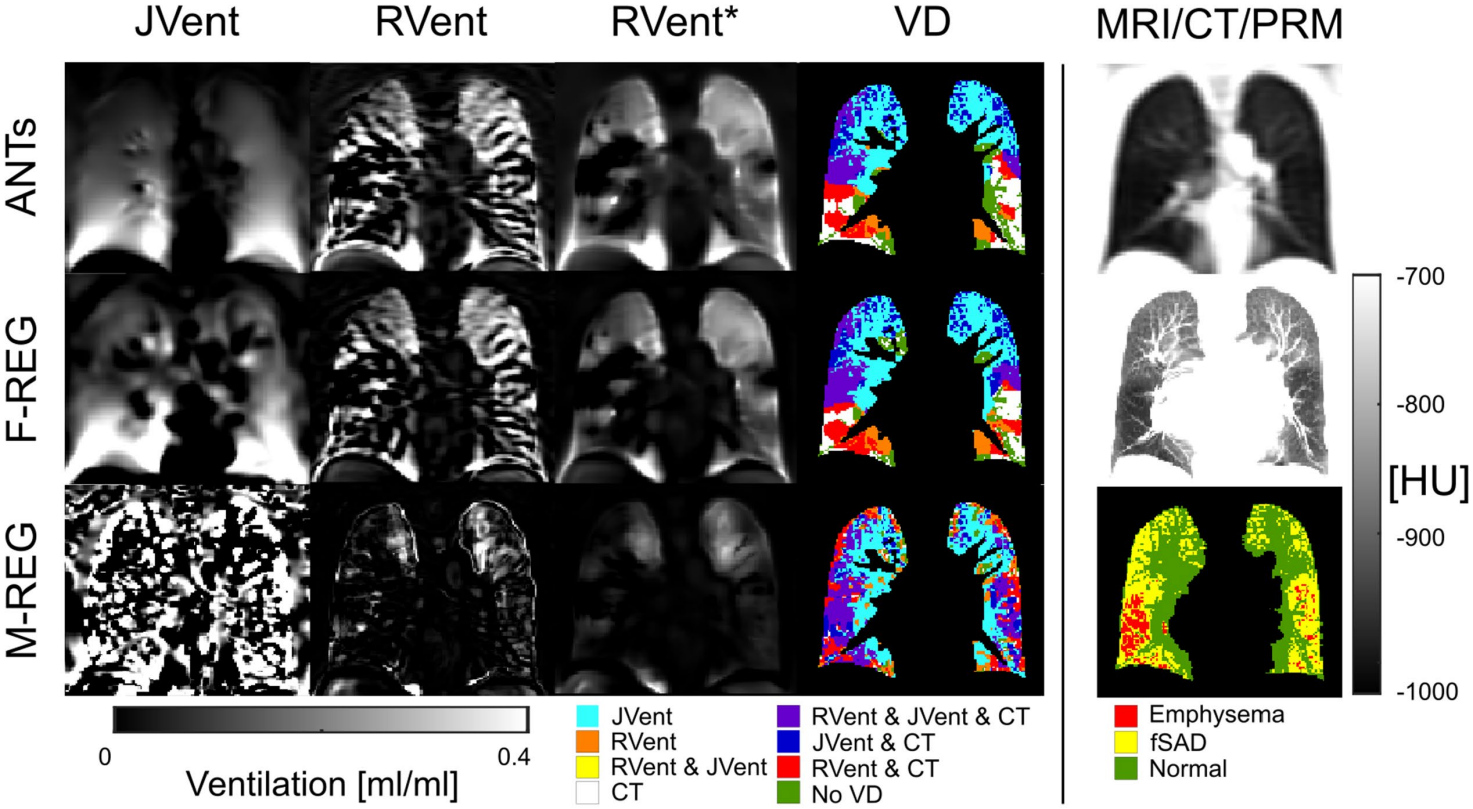
RVent without and with additional filtering (*) in the second and third columns in comparison to Jvent (first column) for a female COPD (GOLD III) patient (age = 63). Rows show the three registration variants. The last column shows the expiration image for anatomical reference, CT in expiration, and parametric response mapping (PRM). The fourth column demonstrates the alignment of the respective ventilation defects derived from RVent* and JVent to PRM. Please note that JVent and RVent show inversed VD patterns: RVent detects VD in the lower lung similar to PRM, while JVent shows mainly unmatched defects in the upper lung regions. ANTs: advanced normalization tools, F-Reg: Forsberg registration, M-Reg: Matlab registration, RVent: regional ventilation, RVent*: filtered regional ventilation, JVent: Jacobian determinant ventilation, PRM: parametric response mapping, fSAD: functional small airway disease, VD: ventilation defect, COPD: chronic obstructive pulmonary disease.

Typical examples of RVent* and JVent maps with PRM and defect distributions are displayed in Figures 7–9. The first shows aligned RVent* and JVent defects with CT in UR/UL quadrants. The second demonstrates a mixed concordance of RVent* and JVent for aligned defects in UR/UL and major CT-aligned RVent defects in LR/LL with no corresponding JVent defects. The third demonstrates a case with CT-aligned RVent defects in LR, LL with no corresponding JVent defects. Contrary to this, JVent shows unaligned major defects in UR/UL. For all cases, the JVent map derived from M-REG was unusable.

## 5 Discussion

This study describes a feasible framework to create synthetic data mimicking a free-breathing lung MRI acquisition. ASYLUM was used to analyze differences between signal- and deformation-based lung ventilation measurements using different registration algorithms. Both the registration algorithms and functional measurement methods showed significantly different results. Overall, M-REG showed incorrect deformation fields, which resulted in unusable JVent, but more or less comparable measurements regarding RVent in comparison to ANTs and F-REG. These registration variants also yielded deformation and JVent, similar to the known REF registration. Nevertheless, further analysis showed a strong regional JVent bias, which resulted in high defect classification in the upper lung regions with very low specificity. Contrary to this, the artificially created ventilation and perfusion defect regions were mostly correctly identified by the signal-based approach. Similar VD detection differences for JVent and RVent were also found in a patient cohort using CT as a gold standard.

Registration algorithms are an important part of functional lung MRI, have numerous parameters for tweaking, and are known to have a great impact on the final results. While registration algorithms are often tested initially with synthetic, but not necessarily realistic data (42, 47), in the realm of lung function, due to missing ground truth, the registration performance is mostly evaluated solely by testing the reproducibility of the final results and by image similarity metrics (e.g., segmented overlap of edges/features measured with Dice or structural similarity index measure) (38, 41). In this regard, the evaluation of a registration algorithm with a known lung deformation using ASYLUM is a novel approach in this specific field. Although M-REG performed as well as F-REG regarding RMSRE, the correlation to the known deformation revealed, that F-REG and ANTs are recreating actually more accurate deformations. Thus, it was not surprising, that all JVent values of M-REG were unusable. Nevertheless, M-REG leads to similar RVent and QA results, which is an indication of the stability of signal-based calculations. Although this seems paradoxical at first, this finding can be explained by the fact that signal-based approaches require only correct registrations of signal groups and not necessarily correct, i.e., physiological sound movements of individual voxels.

Ensuring physiologic deformation vectors requires additional regularization, e.g., in the form of smoothing. Therefore, the degree of regularization might explain the blurred versions of the ANTs and F-REG registration results in comparison to REF. This blurring can explain the difficulty to distinguish defects from the surrounding JVent values. Both registrations also overestimated the movement in the lower lung and underestimated the movement in the upper lung regions, leading to a gradient not present in REF data. Then again, signal-based measurements were able to accurately identify defects, but blurring was also visible when inspecting the VV_Comp_|QV voxels, which were distributed as speckles and therefore are a good indicator for the accuracy of a registration. Registration artifacts at the edges can also be explained by smoothing and were most prominent for ANTs. As expected, the influence of these edge artifacts was prominent when dealing with mean values but disappeared in the median statistic. Defects VD|QD_High_, which were clearly distinguishable from the surrounding parenchyma values, were also more easily identified with JVent. Such regions act as landmarks and probably lead to significant changes upon relocation in the minimization process during registration, resulting in better registration of such regions.

The defect patterns identified in ASYLUM were similarly observed in patients with COPD. Lower lung regions showed much lower defect percentage with JVent in comparison to upper lung regions. In addition, RVent showed defects in the lower lung region with no correspondence in JVent. CT showed higher correspondence of ventilation defects and RVent regarding the number of defects and their regional distribution. Therefore, the observed ventilation defect pattern of RVent and JVent is probably linked to the explanations outlined previously.

The observed strong gradient of decreasing JVent in the superior direction was not reported in studies with hyperpolarized gas MRI, which is considered a gold standard (26, 48) but can be observed in figures showing Jacobian determinant measurements from various studies (8, 10, 11). Similar to findings in our study, Castillo et al. found a good global correspondence of CT HU-derived ventilation in comparison to Jacobian-based methods, but a better correlation of CT HU when comparing on a regional level with SPECT/CT gold standard (49). The less pronounced differences might be explained by the fact that CT is an easier modality to achieve accurate registration as it offers more distinct landmarks due to its higher spatial resolution. Partially different results and interpretations were reported by Tan et al. in a preprinted article involving six healthy volunteers. The authors found a better correspondence of Jacobian-derived ventilation to segmented lung volumes, concluding a less stable performance of signal-based measurements due to low SNR and registration errors (11). While a global better correspondence to lung volumes does not contradict our results, which mainly indicate problems of JVent on a regional level, in contrast, our results suggest a more stable performance of signal-based methods as discussed previously. In fact, registration errors would affect JVent more directly, by definition. Nevertheless, the authors raise a valid point regarding additional signal variations apart from proton density like T2*, which can affect signal-based methods and lead to errors. However, ultimately, both measurements are always intertwined to a certain degree as they depend on each other and result in identical or nearly identical results in theory or when using perfect registration, as demonstrated by ASYLUM. Although the slightly smaller relative differences (except for the edge artifacts) for RVent and QA favored ANTs over F-REG, F-REG was significantly faster. Overall, similar registration performance was observed for both algorithms, as reported by Klimeš et al. (41).

The idea of using digital models for validation is not new, but to the authors’ knowledge, most models stem from radiotherapy and were never employed in the context of functional lung MRI. In general, models can be based on real data (50, 51), be built from scratch (52), or a mixture of both (53–56). Pure patient-based models exhibit the most realistic data but require expert annotation or fiducial markers (57). Completely modeled variants allow for most control but might be too simplified. One of the more recent models, the 4D CT/MRI Breathing Anthropomorphic Thorax (CoMBAT) phantom (56) encompasses realistic movement and tissue parameters (T1, T2, and proton density) using real MR acquisitions and complex modeling of the acquisition and reconstruction side for organ motion quantification and management in image-guided radiotherapy. The CoMBAT model is much more complex than ASYLUM, but it later offers complete control over the regional movement of the different defect classes, which is crucial for this study and post-processing evaluations. Nevertheless, more elaborated models like CoMBAT might be helpful to create more realistic versions of ASYLUM by adapting certain aspects. In addition to the demonstrated application of examining the difference in registration performance with focus on signal and deformation-based ventilation measurements, ASYLUM can be used for completely different aspects, including (1) comparing similar post-processing methods in their performance (e.g., two signal-based approaches) (2), optimizing parameter settings of certain post-processing aspects like filter settings or registration, and (3) testing which minimal defect sizes can be detected with a certain method. Even slight variations of method implementation (e.g., different programming languages) can be validated by sharing ASYLUM results across different sites.

The limitations of this study include the fact that only a limited number of parameter variations were used for this initial study to remain within a reasonable scope. Results may vary depending on the chosen model parameters, such as expansion rate, SNR, defect severity spectrum (e.g., reduced ventilation instead of no ventilation), and defect size. A further limitation is the simplification of the model with regard to lung shape and the isolated one-dimensional movement of the lung. This might be important, as registration is guided by shapes and structures. Additional details in the lung (e.g., vessels) might lead to better registration results. Therefore, the presented results might underestimate the performance of registration, and further improvements of ASYLUM should address this point. On the other side, the 1D movement of ASYLUM should be easier to register in comparison to real motion, which also involves through-plane motion. In summary, both simplifications act as antagonistic factors regarding registration performance. Similarly, increased or decreased SNR will result in more or less accurate registration and parameter results. Based on the necessity to include additional filtering for the real MR data, the SNR was overestimated in the case of ASYLUM. MR sequence parameters, including TE, TR, and MR physics like relaxation, were not modeled with ASYLUM and therefore limit the model’s capability to assess the acquisition aspect and might introduce additional discrepancies to real data. To test the whole capability of ASYLUM, especially in regard to its dynamic components/classes, a phase-sensitive analysis of the whole time series, as in PREFUL, is necessary but was omitted to maintain the concise scope of this study. In addition, registration parameters offer a lot of opportunities for tweaking and can substantially alter the results. For this study, parameters, which delivered visually acceptable results for real data and were used in previous studies were employed. A systematic fine-tuning of parameters with ASYLUM was not performed and might lead to changes in the findings. In addition, models only approximate reality and require some sort of validation. Therefore, validation with real data can never be replaced completely.

Finally, the used gold standard in the patient cohort (CT PRM) is also dependent on registration algorithms and might contain a bias, and defects derived from the emphysema and fSAD classes do not necessarily correspond to ventilation defects. However, as discussed, the CT modality is probably more likely to achieve accurate registration as it offers more distinct landmarks due to its higher spatial resolution. Furthermore, a high correlation of ventilation defects and emphysema and fSAD was found previously (58).

## 6 Conclusion

The digital lung model framework ASYLUM was introduced for the validation of free-breathing functional lung MRI post-processing pipelines. As a first scenario, the influence of registration algorithms, two ventilation methods, and one perfusion quantification method was validated. The findings suggest that JVent, as derived from registration methods and parameters evaluated in this study, leads to a significant bias in the regional ventilation calculation and subsequent defect detection. Analysis of patient data and comparison with CT support these findings. Thus, without extensive registration testing and optimization, the use of JVent would result in unreliable defect classifications not suited for clinical/diagnostic decision-making. In contrast, signal-based regional ventilation assessment was a reliable method in the investigated setting.

## Data Availability

The datasets presented in this study can be found in online repositories. The names of the repository/repositories and accession number(s) can be found at: https://sourceforge.net/projects/asylum-repo/files/.

## Glossary

A: Signal amplitude
ANTs: Advanced normalization tools registration package
ASYLUM: A synthetic lung model
BW: Bandwidth
C: Condition for ventilation compensation
CoMBAT: 4D CT/MRI breathing anthropomorphic thorax
CT: Computed tomography
e: Expansion factor
E: Local expansion matrix
Exp: Expiration
FOV: Field-of-view
F-REG: Forsberg registration package
F_x,y_: Forward deformation field
G: Image geometry
HU: Hounsfield units
i,j: Irregular grid
Insp: Inspiration
I_x,y_: Inverse deformation field
j: Phase
JVent: Deformation-based regional ventilation measurement
LL: Lower-left quadrant
LR: Lower-right quadrant
MBW: Multiple breath wash out
M-REG: A diffeomorphic demons registration algorithm implemented in MATLAB
MRI: Magnetic Resonance Imaging
PREFUL: Phase-resolved functional lung
PRM: Parametric Response Mapping
Q: Perfusion
QA: Signal-based perfusion-weighted amplitude measurement
QD: Perfusion defect
r(t,x): Respiration factor derived from v(t,x)
REF: Reference (known) registration
Reg: Fixed respiration state
RMSRE: Root mean squared relative error
ROI: Region of interest
RVent: Signal-based regional ventilation measurement
s: Signal
S: Scattered interpolant
s(t): signal time-series
SENCEFUL: Self-gated noncontrast-enhanced functional lung
SNR: Signal-to-noise ratio
SPECT: Single-photon emission computed tomography
TE: Echo time
TOF: Time-of-flight effect
TR: Repetition time
UL: Upper-left quadrant
UR: Upper-right quadrant
v: Volume
V: Ventilation
v(t,x): volume surrogate voxel time-series
VD: Ventilation defect
VD|QD_High_: Ventilation and perfusion defect at high signal
VD|QD_Insp_: Ventilation and perfusion defect at inspiration signal
VD|QD_Low_: Ventilation and perfusion defect at signal below inspiration level
VV: Ventilated volume
VV|QD: Ventilated perfusion defect
VV|QV: Normally ventilated and perfused voxels
VV|QV_Delay_: Voxel with normal ventilation but delayed perfusion
VV_Comp_|QV: Compensatory ventilated and normally perfused voxels
VV_Delay_|QV: Delayed ventilation and normally perfused voxels
x,y: Regular grid
•: Warping operator
